# Insight into extracellular vesicles in vascular diseases: intercellular communication role and clinical application potential

**DOI:** 10.1186/s12964-023-01304-z

**Published:** 2023-10-31

**Authors:** Wenxiu Liu, Meiqi Jin, Qiuyan Chen, Qiaoyu Li, Xiaoyan Xing, Yun Luo, Xiaobo Sun

**Affiliations:** 1 Institute of Medicinal Plant Development, Peking Union Medical College and Chinese Academy of Medical Sciences, Beijing, 100193 China; 2 Beijing Key Laboratory of Innovative Drug Discovery of Traditional Chinese Medicine (Natural Medicine) and Translational Medicine, Beijing, China; 3 Key Laboratory of Bioactive Substances and Resource Utilization of Chinese Herbal Medicine, Ministry of Education, Beijing, China

**Keywords:** Extracellular vesicles, Vascular disease, Intercellular communication, Clinical application, Targets, Biomarkers

## Abstract

**Background:**

Cells have been increasingly known to release extracellular vesicles (EVs) to the extracellular environment under physiological and pathological conditions. A plethora of studies have revealed that EVs contain cell-derived biomolecules and are found in circulation, thereby implicating them in molecular trafficking between cells. Furthermore, EVs have an effect on physiological function and disease development and serve as disease biomarkers.

**Main body:**

Given the close association  between EV circulation and vascular disease, this review aims to provide a brief introduction to EVs, with a specific focus on the EV cargoes participating in pathological mechanisms, diagnosis, engineering, and clinical potential, to highlight the emerging evidence suggesting promising targets in vascular diseases. Despite the expansion of research in this field, some noticeable limitations remain for clinical translational research.

**Conclusion:**

This review makes a novel contribution to a summary of recent advances and a perspective on the future of EVs in vascular diseases.

Video Abstract

**Supplementary Information:**

The online version contains supplementary material available at 10.1186/s12964-023-01304-z.

## Background

**Fig. 1:**
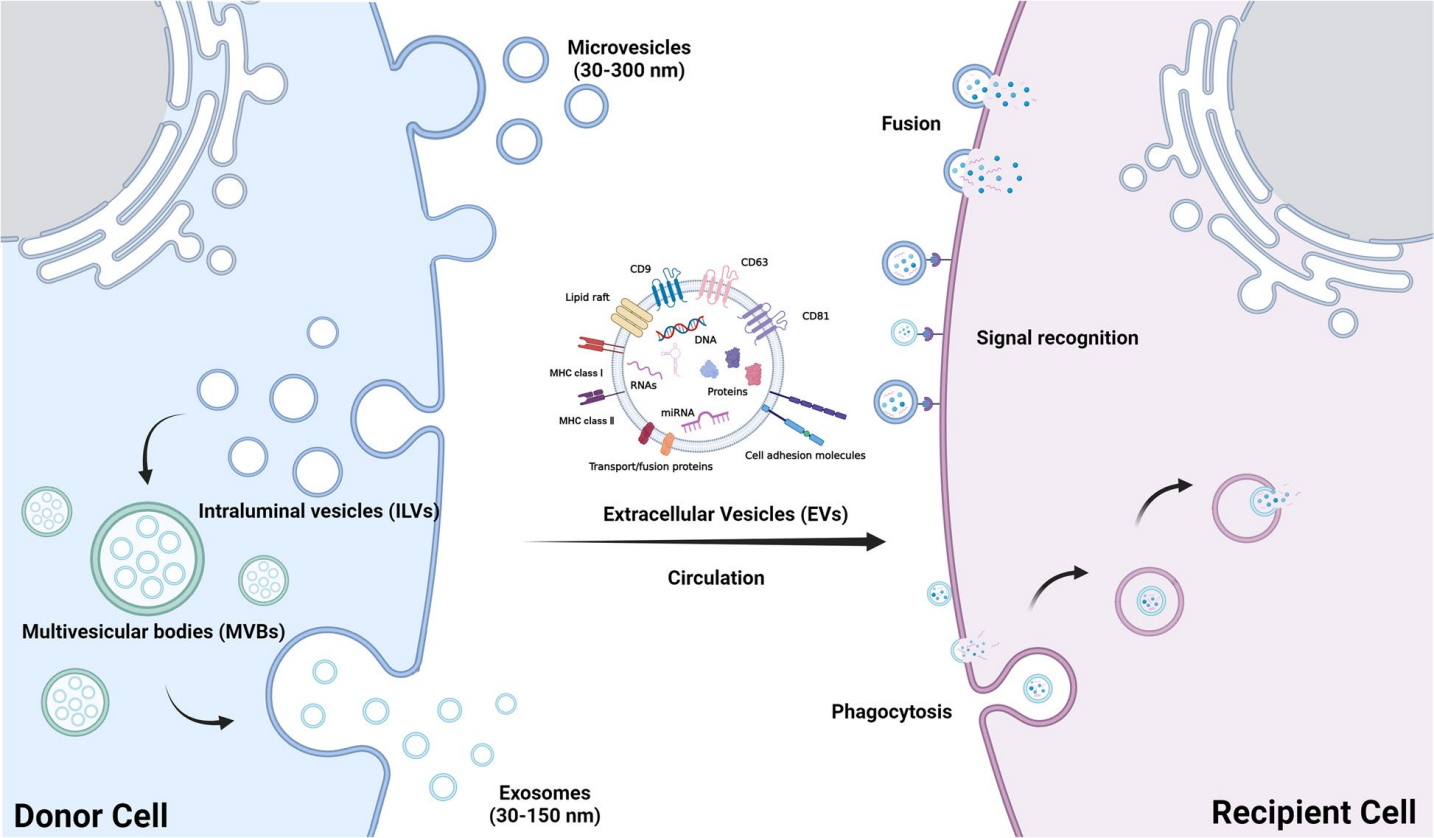
Extracellular vesicle (EV) biogenesis and intercellular communication between donor and recipient cells that EVs mediate Multiple small vesicles originating from the endosome membrane and sprouting inward are defined as intraluminal vesicles (ILVs). Endosomes containing ILVs are called multivesicular bodies (MVBs). The MVBs subsequently fuse with the plasma membrane to release ILVs into the extracellular space to form exosomes. Microvesicles and apoptotic bodies are generally large and can be directly formed by plasma membrane budding. EVs in circulation carry various cargo types, such as CD9, CD63, CD81, and microRNAs, and enter donor cells by fusion, signal recognition, and phagocytosis

Extracellular vesicles (EVs) are vesicles derived from the endosomal system or plasma membrane of nearly all cell types released into the extracellular space. They are a type of lipid bilayer structure without a functional core and replicable capacity [1, 2]. EVs can be classified as exosomes (30–150 nm diameter), microvesicles (30–300 nm diameter), and apoptotic bodies of 1 μm diameter [3–6]. At first, EV secretion was regarded as a means that cells clear useless or harmful components in them [7, 8]. However, with further recent development, it has become apparent that EVs function as a “postman” between donor and recipient cells, transmitting biological information and maintaining intercellular communication [9]. This new understanding of EVs is based on their biogenesis and uptake (Fig. 1) [10]. More importantly, EVs carry multiple bioactive cargoes, such as DNAs, RNAs, proteins, and lipids that contain the information to regulate physiological and pathological processes [11]. Additionally, EVs can serve as carriers for active molecules and exogenous drugs. Encapsulated within lipid bilayers, these biological molecules and medicines are protected from enzymatic degradation and other substances, enabling them to stably exist in circulation [12]. Moreover, EVs can penetrate several physiological barriers, especially the blood–brain barrier (BBB), providing a solution to one of the bottlenecks in treating diseases of the central nervous system (CNS) [13, 14]. These biological characteristics provide great advantages for EVs in intercellular communication. By transmitting biological signals among cells, EVs participate in and affect cell function and state under physiological and pathological conditions. EVs play essential roles in angiogenesis [15], neurogenesis [16], immune response [17, 18], myocardial repair [19], wound healing [20], and other aspects, eventually regulating the disease process.

Vascular diseases are mainly related to vascular lesions affecting the circulatory system, which affect the patients’ quality of life and seriously threaten their life and health [21]. Among these, cardiovascular diseases and cancer rank first and second, respectively, in terms of global mortality. Other vascular diseases such as diabetic complications and acute lung injury (ALI) are also important lethal factors. Various vascular diseases can affect each other. Atherosclerotic plaque rupture often leads to myocardial infarction, stroke, or pulmonary embolism [22]. Hypertension, aneurysms, vascular malformations, hematological diseases, tumors, and other diseases mostly cause cerebral hemorrhage [23]. Vasculopathy and abnormal angiogenesis are normal pathological processes of vascular diseases; however, their distinct pathogenesis remains intricate and has not been thoroughly elucidated [24, 25]. Currently, the methods to prevent and treat vascular diseases are limited. For instance, no immediate and effective treatments exist for ischemic stroke (IS) other than reperfusion through tissue plasminogen activator (tPA) thrombolytic and mechanical thrombectomy therapy; however, its treatment time is exact and it may cause a second cerebral injury [26]. For patients with diabetic foot ulcers (DFU), the current treatment methods are limited to antibiotic administration, blood glucose control, and surgical amputation, with insufficient efficacy or unacceptable results, which brings great trouble to the patients' lives [27]. Emerging evidence has revealed the great potential of EVs in treating vascular diseases.

According to the International Society for Extracellular Vesicles, most studies on EVs mainly focus on their biological function other than their biogenesis process and the specific biomarkers on the EV subtypes have not been determined. Therefore, we have uniformly used the generic term “extracellular vesicle” (EV) in this review [2]. Herein, we provide a summary of the intercellular communication that EVs mediate in vascular diseases, mainly referring to the relevant literature published in the last five years. We discuss the biological roles of EVs (including endogenous and exogenous sources) and their cargoes derived from different cells acting on recipient cells in cerebrovascular diseases, cardiovascular diseases, diabetic complications, tumors, and other vascular diseases. Additionally, the promising uses of EVs as diagnostic and prognostic biomarkers, therapeutic targets, and drug-delivery vehicles for vascular diseases are summarized.

## Biogenesis and Composition of EVs

EVs exist in the biological fluids of organisms after their release, including blood, saliva, and urine [6]. They arrive at the recipient cells via circulation and are internalized through direct signal recognition, membrane fusion, and indirect phagocytosis (Fig. 1) [10]. According to recent studies, two main ways of generating EVs exist. Exosome biogenesis originates from the endosomal system [28]. The endosomal membrane sprouts inward to form multiple small vesicle structures called intraluminal vesicles (ILVs). At this stage, endosomes containing ILVs are defined as multivesicular bodies (MVBs). Subsequently, MVBs fuse with the plasma membrane to release ILVs into the extracellular space to form exosomes [28]. Microvesicles and apoptotic bodies are generally larger than exosomes and can be directly formed by plasma membrane budding [28]. During this process, EVs carry various cargo types inside and on their surface (Fig. 1), which contributes to their heterogeneity. MicroRNAs (MiRNAs) and proteins are the most commonly studied molecules. MiRNAs, with an approximate length of 18–25 nucleotides, are classified as small non-coding RNA [29]. It modulates the expression of downstream target genes in recipient cells by binding to the mRNA transcript 3′ untranslated regions (3′ UTRs) of the target gene [30]. Proteins on the EV surface mainly mediate recognition between EVs and target cells and can be used to identify EVs, seek EV origins, or act as diagnostic markers for diseases, such as tetraspanins (CD9, CD63, and CD81), transport/fusion proteins, cell adhesion molecules, major histocompatibility complex class I (MHC I), and MHC class II (MHC II). (Fig. 1) [31]. Nevertheless, proteins such as the fibronectin type-III domain-containing protein 5 (FNDC5)/irisin [32], angiotensin-converting enzyme 2 (ACE 2) [33], and matrix metalloproteinase 9 [34], packaged in bilayer lipid membranes primarily carry communication information and affect biological functions of recipient cells. And they can also be regarded as characterization markers, including ALG-2 interacting protein X, tumor susceptibility gene 101, and vacuolar protein sorting 4 [2, 28]. These cargoes play a vital role in intercellular communication that EVs mediate.

## Intercellular Communications that EVs Mediate in Vascular Diseases

### Cerebrovascular Disease

#### Stroke

Stroke is an acute cerebral disease caused by sudden bleeding or blood vessel blockage and is the second most common cause of death and the third leading cause of disability [35]. It is classified into two types: IS and intracerebral hemorrhage (ICH) [36].

#### Ischemic Stroke (IS)

Increasing evidence supports the potential of EV-based therapeutics in recovering brain function after IS. These therapeutics mediate intercellular communication in the pathological process, mainly involving cells (neurons, astrocytes, microglia, and oligodendrocytes) originating from the CNS and stem cells [37, 38]. For example, neuron-derived EVs containing miR-98 can be delivered from the neurons to microglia, and miR-98 has been shown to protect ischemia-induced stress-but-viable neurons from microglial phagocytosis by targeting the platelet-activating factor receptor [39]. This further confirmed the neuroprotective effect of miR-98 after cerebral ischemia. Microglia, divided into M1 inflammatory and M2 anti-inflammatory phenotypes, perform immunological functions in the CNS, and microglia-derived EVs regulate inflammation and glial survival [37]. Raffaele et al. demonstrated that infusing EVs derived from pro-regenerative microglia maintained the normal function of microglia/macrophages and promoted oligodendrocyte precursor cell (OPC) maturation in the penumbra in mice with permanent middle cerebral artery occlusion (MCAO) [40]. Additionally, EVs derived from M2 microglia improved sensorimotor and cognitive function by enhancing oligodendrocyte generation and white matter repair via miR-23a-5p, which has been shown to facilitate oligodendrogenesis, possibly by directly targeting OLIG3-oligodendrocyte transcription factor 3 after cerebral ischemia [41]. EVs from M2 microglia mitigated glial scar formation and repressed astrocyte activation to improve neuronal regeneration in mice undergoing MCAO surgery. This process depends on the increase in miR-124 in the ischemic mouse brain through EVs, which block the signal transducer and activator of transcription 3 signal pathway [42]. Interestingly, EVs released from astrocytes play a neuroprotective role in most cases [43]. Intracerebroventricular injection of astrocyte-derived EVs accelerated spontaneous recovery in MCAO rats, promoting axonal growth and neuronal survival [44]. However, Hira et al*.* demonstrated that exosomes derived from activated astrocytes have detrimental effects, including inhibition of axonal growth. Further research found that a semaphorin 3A inhibitor could decrease astrocyte activation and reduce exosome release, providing a promising strategy to improve functional recovery after ischemia [45].

Intercellular communication mediated by exogenous EVs from stem cells is also vital after IS, according to previous studies that have demonstrated the role of EVs in neuroprotective effects and neuroimmunological regulation [38]. MiR-25-3p enriched in EVs from adipose-derived mesenchymal stem cells (MSCs), degrades p53 mRNA and downregulates p53 and Bcl-2 interacting protein 3 levels, eventually decreasing autophagy levels in neurons [46]. Qiu et al*.* reported that MSC-derived EVs (MSC-EVs) repressed the inflammation response and astrocyte activation via miR-125b-5p / toll-like receptor 4 (TLR4) / nuclear transcription factor-kappaB (NF-κB) signaling pathway to protect BBB integrity against IS [47]. Interestingly, EVs derived from embryonic stem cells (ESCs) can alleviate neuroinflammation by activating the TGF-β/Smad signaling pathway to increase the number of regulatory T cells, which inhibit the inflammatory response after stroke [48]. However, the specific cargo of this EV type requires further investigation.

#### Intracerebral Hemorrhage (ICH)

ICH is commonly caused by hypertension, which results in nontraumatic parenchymal vascular hemorrhage [35]. In recent years, EV studies on this type of cerebral disease have focused on stem cells with regenerative functions [49]. For instance, Han et al. confirmed that multipotent mesenchymal stromal cell-derived exosomes enhanced functional recovery after ICH in rats by promoting neurogenesis and angiogenesis; however, the underlying mechanism was not revealed [50]. Bone marrow mesenchymal stem cells (BMSCs) are a popular stem cell source for exosomes in treating ICH [49]. BMSC-exosomal miR-23b reduced oxidative stress and pyroptosis in ICH rat brains and alleviated brain damage caused by cerebral hemorrhage. Further in vitro studies have shown that miR-23b represses microglia/macrophage oxidative stress and the NOD-like receptor family pyrin domain containing 3 (NLRP3) inflammasome-mediated pyroptosis by regulating the expression of phosphatase and tensin homolog deleted on chromosome 10 (PTEN) [51]. This provides a novel approach for delving into the role of exosomes in pyroptosis after ICH. Similarly, exosomes from adipose-derived stem cells (ADSCs) effectively improved the neurological function of ICH mice, whose enriched miR-19b-3p directly inhibited the iron regulatory protein 2 to alleviate neuronal ferroptosis [52]. Additionally, exosomes containing the signal regulatory protein α (SIRPα) variants from BMSCs can modulate microglia/macrophage polarization by blocking CD47 and SIRPα interaction, which provides a target to promote microglial phagocytosis of the red blood cells to accelerate hematoma removal [53]. Other than stem cells, EVs derived from activated microglia promote neuronal necroptosis through transferring miR-383-3p, which decreases activating transcription factor 4 expression in vitro. Additionally, miR-383-3p is upregulated in rats with ICH and exacerbates cerebral hemorrhage injury in vivo, providing a promising therapeutic target for ICH [54].

Most studies on ICH have used EVs isolated in vitro and injected into ICH animal models or co-cultured with cell models. Interestingly, a recent study indicated that endogenous exosomes exert a beneficial effect on neuroinflammation suppression by injecting an inhibitor of exosome generation and release (GW4869) into ICH mice, which also proves that exosomes act as a bridge between the brain and immune system in ICH mice [55].

#### Vascular Dementia (VD)

VD, the second most common dementia after Alzheimer’s disease (AD), is a common type of vascular cognitive impairment with cognitive dysfunction, mainly caused by cerebrovascular lesions [56, 57]. EVs play a vital role in VD pathogenesis. For instance, increased miR-154-5p expression induces endothelial progenitor cell (EPC) dysfunction and inhibits angiogenesis in VD [58]. Additionally, Ma et al. found that miR-132-3p expression is markedly decreased in the brain tissue of VD mice, and treatment with miR-132-3p from MSC exosomes can improve neuronal injury, synaptic dysfunction, and cognitive deficits [59]. Furthermore, miR-132-3p inhibited the expression of ribosomal arginine synthetase 1 and activated the Ras/Akt/GSK-3β pathway to restore neurite growth and synaptic density [59]. Additionally, hippocampal neural stem cells (HNSCs) are important for maintaining cognitive function, and their impairment and senescence can lead to cognitive decline [60]. Interestingly, ESC-derived EVs contribute to lysosomal activation by transporting high levels of miR-17-5p, miR-18a-5p, miR-21-5p, miR-29a-3p, and let-7a-5p, thereby rejuvenating aging HNSCs [61]. Besides miRNAs, myocardial infarction- associated transcript (MIAT) from HNSC exosomes ameliorates hippocampal neuronal cell damage by inhibiting miR-34b-5p; however, whether MIAT improves cognitive impairment by inhibiting miR-34b-5p requires further verification [62]. Collectively, intercellular communication that EVs mediate plays an important role in VD.

#### Cerebral Small Vessel Disease (CSVD)

CSVD results from microangiopathy of the brain [63]. Research on the role of EVs in CSVD has only recently emerged. MSC-EVs decrease IL-1β and IL-6 expression in lipopolysaccharides-stimulated BV2 cells and attenuate inflammation [64]. Researchers built animal models of CSVD using salt-loaded SBH/y rats to induce hypertension and found that cognitive deficits improved after MSC-EV treatment [64]. However, the underlying mechanisms require further in-depth studies [64]. Most studies have focused on EVs from the serum or plasma of patients and healthy volunteers as diagnostic markers for CSVD; however, a large gap exists in the understanding of the pathological roles and therapeutic potential of EVs in this disease. Seeking donor and recipient cells of EVs will help to further understand CSVD pathogenesis and discover new therapeutic targets.

**Fig. 2:**
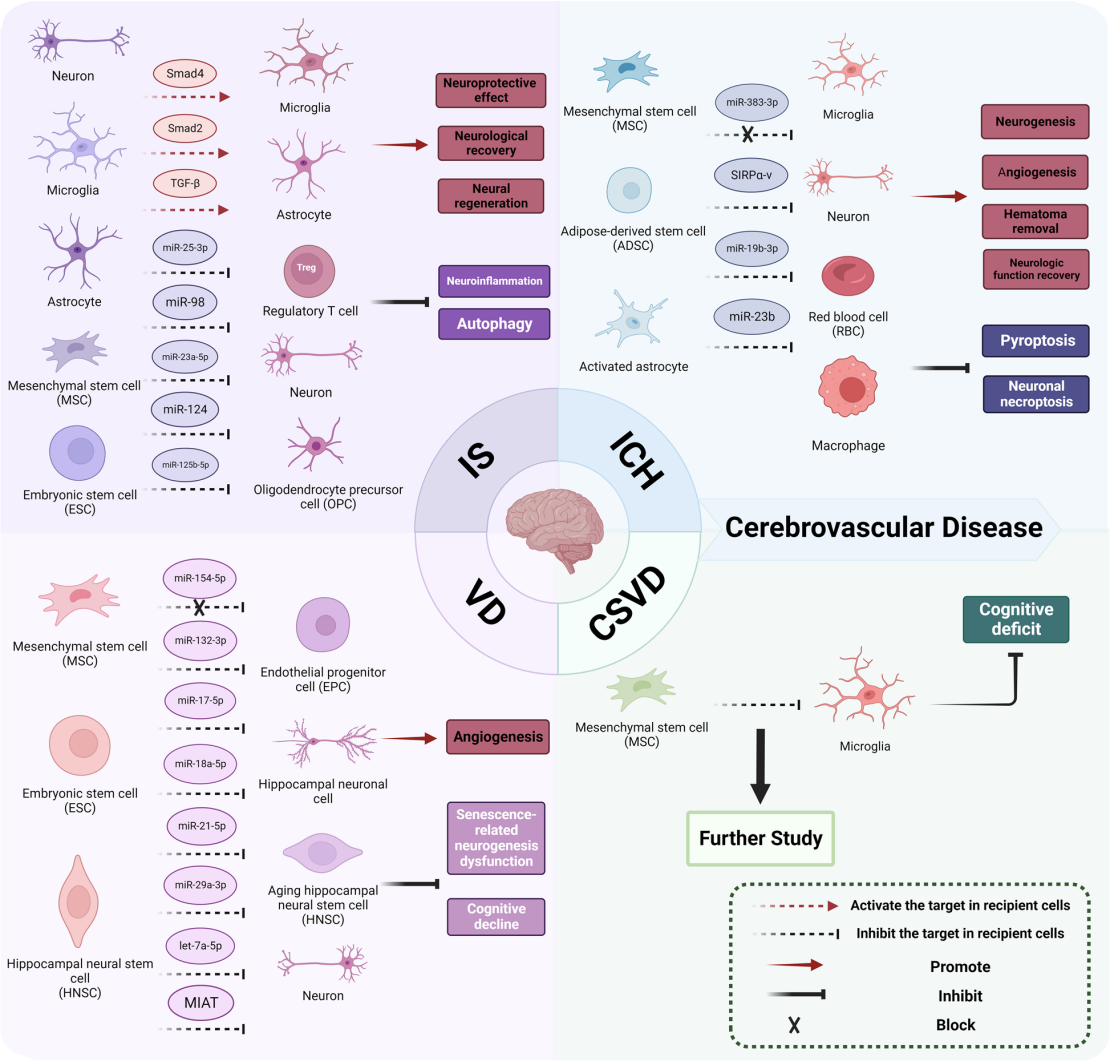
Intercellular communication that EV cargoes mediate regulates the cerebrovascular disease progression Cargoes from different donor cells, including neurons, microglia, astrocytes, mesenchymal stem cells (MSCs), embryonic stem cells (ESCs), adipose-derived stem cells (ADSCs), and hippocampal neural stem cells (HNSCs) can activate or inhibit specific targets in recipient cells during cerebrovascular disease progression, including ischemic stroke (IS), intracerebral hemorrhage (ICH), vascular dementia (VD), and cerebral small vessel disease (CSVD). These processes promote or inhibit the physiological and pathological processes of cerebrovascular diseases, and blocking the delivery of miR-383-3p and miR-154-5p can improve ICH and VD. Further studies on intercellular communication that EV cargoes mediate in CSVD are needed

Intercellular communication that EVs mediate in cerebrovascular diseases is specifically shown in Fig. 2.

### Cardiovascular Disease

#### Hypertension

Hypertension, the main cause of cardiovascular disease morbidity and mortality, refers to a systolic blood pressure of at least 130 mmHg or a diastolic blood pressure of at least 80 mmHg [65]. EVs play an important role in blood pressure regulation. EVs isolated from normotensive Wistar–Kyoto rats reduced endothelium-dependent vasodilation, possibly through nitric oxide synthase-dependent mechanisms; however, EVs isolated from spontaneously hypertensive rats changed the function of endothelium-mediated vascular tone in the resistance artery, which implies that EVs are influential in hypertension development [66]. EVs and their contents that mediate cell–cell communication are promising treatment tools [67].

The vascular wall is mainly composed of endothelial cells (ECs) and subendothelial smooth muscle cells (SMCs). Vessel wall remodeling and endothelial dysfunction related to abnormal EC-SMC communication are important in hypertension [68, 69]. Wang et al. provided evidence that EVs from damaged ECs are transported to SMCs, causing arterial remodeling. Inhibiting miR-320d/423-5p expression in EVs alleviates hypertension progression in vivo, and these two miRNAs could be used as biomarkers for predicting arterial remodeling [70]. MiR-483-3p in EVs from ECs exerts protective effects against endothelial dysfunction [71]. Shang et al. speculated that miR-483-3p increased in ECs at the beginning of hypertension and was transported to SMCs to prevent aortic stiffness; however, with hypertension progression, miR-483-3p level decreased owing to disruption of vessel homeostasis [71]. Additionally, EVs containing angiotensin-converting enzyme ACE, miR-155-5p, and miR-135a-5p secreted by adventitial fibroblasts play vital roles in regulating vascular remodeling during hypertension progression [72–74]. MiR-155-5p inhibits vascular smooth muscle cell (VSMC) proliferation and remodeling by targeting ACE, whereas miR-135a-5p promotes VSMC proliferation and hypertension by repressing the expression of FNDC5 [72–74].

Interestingly, stem cell-derived EVs improve age-related vascular endothelial dysfunction and contribute to antihypertension; however, the components of this type of EVs are still unclear [75].

#### Atherosclerosis

The pathological process of atherosclerosis, a leading contributor to cardiovascular and cerebral disease mortality, primarily involves ECs, monocytes, macrophages, platelets, and SMCs [76, 77]. These cells interact with each other via EVs and their components to modulate atherosclerosis pathogenesis. ECs deliver EVs carrying miR-92a to macrophages, regulating Krüppel-like factor 4 (KLF4) expression. This promotes the pro-inflammatory response of macrophages and accelerates the uptake of low-density lipoproteins by macrophages, thereby aggravating atherosclerosis [78]. Notably, other than EVs secreted by single cells, monocytes/platelets can also release EVs to activate ECs and play a pro-inflammatory role, which exacerbates atherosclerosis [79]. MSCs mitigate the pathological processes of atherosclerosis by secreting EVs. For example, EVs carrying miR-221 shuttle from human BMSCs to human arterial SMCs and counteract atherosclerotic plaque formation [80]. Additionally, MSC-EVs with high miR-146a levels alleviated angiogenesis to rescue oxidative stress-induced EC senescence [81].

Notably, intercellular communication that EVs mediate can be influenced by the inducible factors of atherosclerosis. A recent study demonstrated an increase in the expression level of miR-27b-3p in serum exosomes and exosomes in the visceral adipocytes of obese mice and patients with atherosclerosis. The downregulation of peroxisome proliferator-activated receptor α in ECs mediated by miR-27b-3p from visceral adipocyte EVs is an important driver of obese endothelial inflammation, which in turn leads to atherosclerosis [82]. Interestingly, smoking is a risk factor for atherosclerosis, and recent studies have shown that nicotine in cigarettes promotes atherosclerosis development by mainly affecting EV-mediated communication between inflammatory and endothelial cells [77]. EVs containing miR-155 from nicotine-stimulated monocytes accelerated endothelial dysfunction and promoted plaque formation [83]. Furthermore, exosomal miR-21-3p from nicotine-induced macrophages facilitated VSMC proliferation and drove atherosclerosis by inhibiting PTEN [84]. Exploring the effects of nicotine on the function of EVs in atherosclerosis has attracted the interest of researchers worldwide.

#### Acute Myocardial Infarction (AMI)

Intercellular communication mediated by EVs is crucial for cardiac repair after AMI in multiple cell types, including cardiomyocytes, ECs, fibroblasts, and immunocytes. Cardiomyocytes and ECs increase EV production after AMI. These EVs accumulate in the ischemic myocardium and are absorbed by infiltrating monocytes to regulate inflammation [85]. Nevertheless, exosomes from infarcted cardiomyocytes can be transferred to bone marrow monocytes, targeting the C-X-C chemokine receptor type 4 (CXCR4) of monocytes to mobilize the progenitor cells via circulation, contributing to ischemic tissue repair. This function relies on miRNAs of damaged cardiomyocyte exosomes; however, which miRNA plays a role requires further exploration [86]. CircUbe3a is a circular RNA (circRNA) found in M2 macrophage-derived small extracellular vesicles (sEVs) that are released into cardiac fibroblasts accompanied by M2 macrophage infiltration during AMI progression. It targets the miR-138-5p/RhoC axis and markedly decreases myocardial fibrosis after AMI [87]. Angiogenesis is a critical process during cardiac healing. Exosomes containing miR-155 released by M1 macrophages are internalized by ECs, inhibiting angiogenesis and ultimately preventing cardiac repair. Five target genes (RAC1, RAK2, Sirt1, Enos, and AMPKa2) of miR-155 were screened using gene sequencing, bioinformatics, and further validation [88]. Thus, miR-155 inhibition may be a novel mechanism for promoting cardiac repair following AMI treatment.

EVs produced by stem cells are innovative cell-free avenues for AMI therapy that effectively function in AMI progression by mediating intercellular communication, especially with MSCs [89]. Xiao et al. provided evidence that the benefits of MSC treatment after AMI were at least partially attributable to the released exosomes containing miR-125b-5p, which reduced autophagic flux [90]. EVs produced by MSCs promote cardiac healing via their contents after the infarction. The levels of miR-486-5p are increased in hypoxia-preconditioned MSC-derived EVs, and this EV type promotes angiogenesis by targeting matrix metalloproteinase 19 in fibroblasts and inhibiting cleavage of vascular endothelial growth factor A (VEGFA). In rodent and nonprimate MI animal models, sEVs containing miR-486-5p have been shown to enhance angiogenesis and improve cardiac function. Importantly, it does not increase arrhythmia incidence, indicating the safety of EV treatment [91]. Intrapericardially injected MSC-derived exosomes are taken up by MHC-II antigen-presenting cells and activate Foxo3, which dominates IL-10, IL-33, and IL-34 expression in antigen-presenting cells, and further activates immunosuppressive regulatory T cells to promote cardiac repair [92]. Interestingly, constructing acellular cardiac scaffolds enriched with MSC-derived EVs increased the neovascularization density and decreased myocardial fibrosis, eventually promoting myocardial repair in an MI pig model [93].

#### Myocardial Ischemia–Reperfusion Injury (MIRI)

EVs are mostly involved in all processes of MIRI, such as the inflammatory response, angiogenesis, and cardiomyocyte survival [94]. Cardiomyocytes, ECs, stem cells, immunocytes, adipocytes, and other cells secrete EVs in this pathophysiology. For instance, EVs from cardiomyocytes enhance endothelial nitric oxide synthase activity in cardiac microvascular endothelial cells (CMECs), which elevates the survival of cardiomyocytes and CMECs, thereby attenuating MIRI [95]. Endothelial-derived EVs improve cardiomyocyte viability by delivering cardioprotective protein cargo to injured cardiomyocytes, ultimately rescuing human laminar cardiac tissues exposed to ischemia–reperfusion injury [96]. Additionally, krüppel–like factor 2 (KLF2), which is highly expressed in ECs, is crucial in inflammation. EVs from KLF2-overexpressing ECs inhibited Ly6Chigh monocyte recruitment by transmitting miR-24-3p, eventually improving the inflammatory response and alleviating MIRI [97]. This study provides a basis for developing therapeutic agents against MIRI. Interestingly, miR-130b-3p, enriched in dysfunctional adipocyte-derived EVs, downregulates the expression of AMP-activated protein kinase α, aggravating cardiomyocyte apoptosis, prejudicing cardiac function recovery, and exacerbating diabetic MIRI [98]. Contrastingly, Crewe et al. showed that adipocytes respond to mitochondrial stress caused by obesity by rapidly releasing EVs containing damaged mitochondria. These EVs enter the circulation and are absorbed by cardiomyocytes, triggering responses of reactive oxygen species that lead to compensatory oxidative stress signaling in the heart, thereby protecting the cardiac tissue from acute oxidative stress and eventually favoring the prevention of MIRI [99]. This suggests that EVs secreted by the same cell type play different or even opposite roles in intercellular communication during MIRI.

Notably, EVs from ADSCs promote cardiac regeneration via miR-210 targeting multiplexed targets. For example, miR-210 promotes angiogenesis in hypoxic ECs by inhibiting Ephrin A expression [100]. Additionally, this miRNA exerts an anti-apoptotic effect on the hypoxic H9c2 cells (a type of rat cardiac myocyte). This study revealed the multiplexed targeting effects of miR-210 present in stem cell-derived EVs [100]. Studies on the intercellular communication mediated by EVs derived from stem cells in MIRI are limited, which requires further exploration.

#### Heart Failure (HF)

HF is the terminal stage of nearly all kinds of cardiovascular diseases and the cardiovascular diseases mentioned above, including hypertension, atherosclerosis, AMI, and MIRI, can eventually lead to HF [101]. Intercellular communication that EVs mediate may be potentially effective in preventing HF in the early stages and ameliorating the final pathological symptoms to repair the injured heart. For instance, EVs enriched with miR-378 secreted from cardiomyocytes under overloaded conditions inhibit excessive fibrosis of cardiac fibroblasts through the paracrine process. Furthermore, miR-378 can target mitogen-activated protein kinase kinase 6 to partially inhibit the mitogen-activated protein kinase signaling pathway. This implies that miR-378 is an endogenous protective factor in the early myocardial hypertrophy stages after mechanical stress and can prevent HF [102]. Additionally, miR-21-5p is dysregulated in exosomes derived from cardiac stromal cells of patients with HF, which impedes cardiac repair. Preclinical studies have shown that miR-21-5p can enhance angiogenesis and cardiomyocyte survival by regulating the PTEN/Akt signaling pathway, indicating that miR-21-5p has great potential in HF treatment [103].

Doxorubicin is a common agent used to build HF animal models and an effective antitumor drug in clinical settings; however, its cardiotoxicity should not be overlooked [104]. Interestingly, stem cell-derived EVs can protect against doxorubicin-induced HF. Human trophoblast exosomes inhibit the expression of miR-200b and increase the level of zinc finger E-box binding homeobox 1, which reduces cardiomyocyte apoptosis and ultimately mitigates HF that doxorubicin induces; however, the specific cargo in exosome functions still needs further exploration [105]. EVs produced by human umbilical cord MSCs suppress oxidative stress and AC16 cell apoptosis mediated by doxorubicin, and their cargo, miR-100-5p, targets NADPH oxidase 4, which plays a cardioprotective role in HF [101]. Additionally, ESC-derived exosomes relieve cardiomyocyte pyroptosis and cardiac remodeling caused by doxorubicin, eventually attenuating its cardiotoxicity [106]. Taken together, the intercellular communication that EVs mediate exerts prominent effects in alleviating HF resulting from doxorubicin treatment, suggesting that simultaneous doxorubicin and EV administration may reduce antitumor drug cardiotoxicity.

**Fig. 3:**
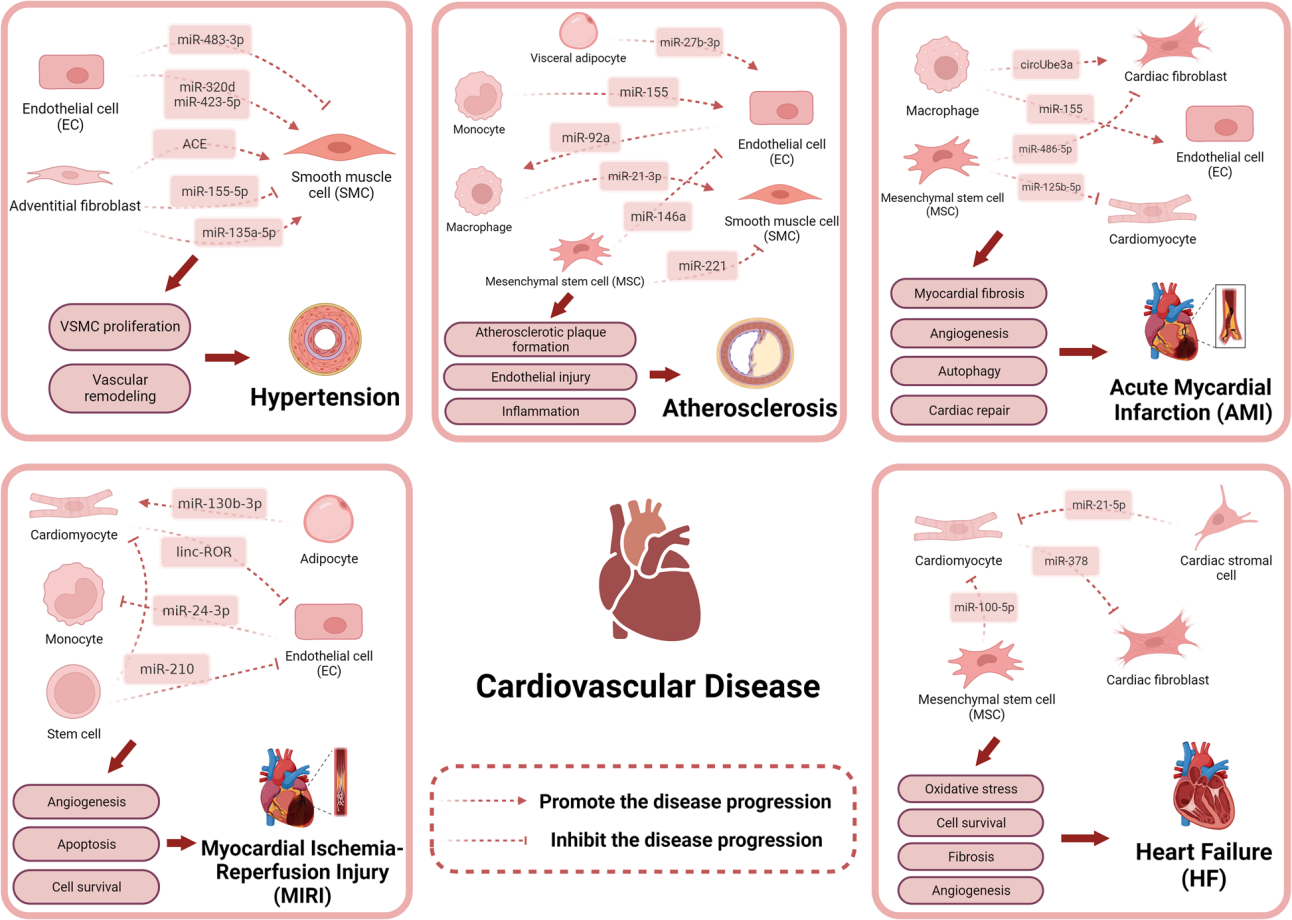
Intercellular communication that EV cargoes mediate regulates cardiovascular disease progression Cargoes from different donor cells, including endothelial cells (ECs), adventitial fibroblasts, monocytes, macrophages, cardiomyocytes, and stem cells can activate or inhibit specific targets in recipient cells during cardiovascular disease progression, including hypertension, atherosclerosis, acute myocardial infarction (AMI), myocardial ischemia–reperfusion injury (MIRI), and heart failure (HF). These processes affect cell proliferation, apoptosis, vascular remodeling, angiogenesis, inflammation, and fibrosis in cardiovascular diseases

Intercellular communication that EVs mediate in cardiovascular diseases is briefly depicted in Fig. 3.

### Diabetic Complications

#### Diabetic Nephropathy (DN)

In the pathological process of DN, EVs from abnormal cells aggravate kidney injury. EVs derived from hyperglycemia-induced activated platelets act on the mammalian target of rapamycin (mTOR) pathway in glomerular ECs to exacerbate glomerular endothelial damage and albumin leakage in urine, promoting the development of DN at an early stage [107]. Additionally, high levels of miR-19b-3p in urine exosomes are associated with the severity of tubulointerstitial inflammation in DN patients. MiR-19b-3p in exosomes can mediate the communication between damaged tubular epithelial cells (TECs) and macrophages. This promotes pro-inflammatory activation of macrophages and drives tubulointerstitial inflammation [108]. Jiang et al. proved the formation of a negative feedback regulation signaling pathway between TECs and macrophages. Lipotoxic TEC-derived EVs activated macrophages and drove them to secrete EVs, promoting apoptosis in TECs damaged by lysophosphatidyl choline [109]. Interestingly, a study reported that miR-199a-5p in EVs secreted by renal proximal TECs exposed to excessive albumin promoted M1 macrophage polarization by targeting Klotho, a protein that polarizes M1 macrophages into M2 and improves renal fibrosis [110]. Additionally, EVs involved in DN pathophysiology also play a protective role. Exosomes secreted by M2 macrophages, for example, inhibit lipopolysaccharides-induced podocyte injury and apoptosis in vitro by miR-93-5p targeting TLR4 in podocytes, protecting kidneys from damage [111].

Emerging evidence has shown that EVs secreted by stem cells function as renal protective preparations in DN. In a streptozotocin-induced rat model, Ebrahim et al. demonstrated that exosomes derived from MSCs alleviated kidney injury via targeting mTOR to promote autophagy [112]. The cargoes transferred from stem cells to recipient cells are closely associated with the protective effects of DN. For instance, miR-486 is downregulated in DN patients, and miR-486 from ADSC exosomes has been proven to protect podocytes against injury in vivo and in vitro by activating autophagy via the Smad1/mTOR signaling pathway [113]. 14–3-3ζ in human umbilical cord MSC-derived EVs inhibits the expression of yes-associated protein to elevate podocyte autophagy activity, which reduces podocyte apoptosis and lessens renal injury [114]. Taken together, EVs derived from stem cells have enormous potential for DN prevention and treatment, especially in regulating autophagy activity.

#### Diabetic Retinopathy (DR)

In recent years, studies on EVs in DR have focused on retinal endothelial cells (RECs), retinal Muller cells, pericytes, retinal microvascular endothelial cells (RMECs), retinal pigment epithelium (RPE), and MSCs. Exosomes collected from the vitreous of proliferative DR patients promoted neovascularization [115]. Further studies revealed that miR-9-3p in exosomes from retinal Muller cells under high glucose conditions targeted sphingosine-1-phosphate receptor 1 in RECs to promote abnormal angiogenesis and aggravate DR [115]. It implies that modulating cell crosstalk is an effective method to inhibit abnormal angiogenesis and repress DR progression. Interestingly, pericyte loss is a well-known feature of DR. The expression of circEhmt1 in exosomes is up-regulated in hypoxic preconditioned pericytes, which protects ECs from injury induced by high glucose by modulating the NFIA/NLRP3 pathway [116]. This improves retinal microvascular dysfunction caused by diabetes. It offers a potential therapeutic target for DR treatment.

EVs secreted by MSCs inhibit the inflammation response, angiogenesis, and apoptosis by shuttling between cells to repress DR progression. He et al. found that EVs secreted from MSCs transferred miR-30c-5p to target phospholipase C gamma 1 in RECs, eventually attenuating inflammation [117]. Additionally, MSC-derived EVs protected RPE cells from high glucose-induced apoptosis and oxidative damage, which was attributed to the cargo called neuronal precursor cell-expressed developmentally downregulated 4 [118]. Intravitreally injecting MSC-EVs into streptozotocin-induced diabetic rats protects the retina and prevents DR development [118]. Under high glucose conditions, EVs derived from adipose MSCs counteract RPE cell apoptosis, Muller cell activation, and RMEC proliferation. MiR-192 carried in this kind of EV negatively regulates integrin alpha 1 to alleviate inflammation and angiogenesis in DR [119]. Human RMECs damaged by high glucose can also be regulated by MSC-EVs. MSC-EVs reduce the inflammation in the retina of diabetic rats, and further study finds that miR-18b contained in MSC-EVs plays an anti-inflammatory and anti-apoptotic role in RMECs stimulated by high glucose [120].

Currently, there are many studies about EVs derived from MSCs mediating intercellular communications during DR development. However, there are few studies on EVs secreted by other cells involved in the pathological process of DR, and further research is still needed.

#### Diabetic Cardiomyopathy (DCM)

DCM is a kind of cardiomyopathy that is not explained exactly by hypertension, ischemic cardiomyopathy, or other common reasons for myocardial tissue lesions in diabetic patients [121]. At present, there are fewer studies about DCM-related EVs compared with other diabetic complications, and most researchers concentrate on cardiomyocytes and cardiac fibroblasts as EV receptors. Senescent cells influence exosomal biogenesis and regulate autophagy flux, ultimately driving the progression of vascular diseases [122]. A study demonstrated that EVs mediated the crosstalk between senescent adipose and cardiomyocytes, and removing senescent adipose alleviated DCM symptoms in streptozotocin type 1 diabetic mice [123]. Mechanistically, inhibiting Rictor expression in cardiomyocytes is how miR-326-3p from senescent adipocyte-derived EVs leads to mitochondrial dysfunction and cardiac diastolic failure [123]. However, this study does not clarify the specific subtypes of senescent cells, and more accurate targets of interaction might be discovered in future research. According to studies on EVs released by CMECs, mammalian sterile 20-like kinase 1 (Mst1) is a detrimental signal in EVs from CMECs to cardiomyocytes that inhibits autophagy, promotes apoptosis, and impedes recipient cell glucose metabolism [124]. Endothelial-specific Mst1 transgenic mice perform poorly in cardiac function and insulin resistance as compared to non-transgenic mice [124]. Interestingly, cardiomyocyte-derived EVs can also act on cardiomyocytes. In high glucose-induced cardiomyocytes, ticagrelor-pretreated cardiomyocyte-derived EVs reduce the expression of biomarker proteins about autophagy, apoptosis, and ER stress [125]. Additionally, after administration of these EVs, down-regulated expression of miR-499, miR-133a, and miR-133b is reversed in cardiomyocytes under diabetic conditions [125].

Acting on cardiac fibroblasts is also an important method to treat DCM. Exosomes secreted by cardiac parasympathetic neurons blunted apoptosis and elevated cardiac fibroblast viability in high glucose conditions [126]. EVs can also build a bridge for interaction between CMECs and cardiac fibroblasts. Exosomes from CMECs containing TGF-β1 mRNA activated cardiac fibrosis in hyperglycemia and promoted myocardial fibrosis in diabetic mice [127]. This study provides a potential method for preventing DCM development. However, the precise underlying mechanism still needs further study.

#### Diabetic Foot Ulcers (DFU)

EVs show great potential for wound healing in DFU. ADSCs promote wound repair by transferring bioactive molecules via EVs in hyperglycemic conditions. ADSCs secreted exosomes with overexpression of nuclear factor-erythroid factor 2 (Nrf2), which enhanced the proliferation and angiogenesis of endothelial progenitor cells (EPCs), markedly reducing the ulcer area in diabetic rats [128]. Another study showed that exosomes produced by linc00511-overexpressing ADSCs inhibited Twst1 ubiquitination induced by progestin and adipoQ receptor 3, which mechanically rescue Twst1 levels in EPCs to improve DFU [129]. ECs, as a key component of angiogenesis, are also regulated by EVs generated from ADSCs. Mmu_circ_0001052 in exosomes from ADSCs suppresses the expression of miR-106a-5p in human umbilical vein endothelial cells (HUVECs), ultimately promoting angiogenesis and wound healing in DFU [130]. In addition, exosomal lncRNA H19 from MSCs protects fibroblasts against apoptosis and inflammation by binding with miR-152-3p to target PTEN. Injecting these exosomes into DFU mice ameliorates wound ulcers [131]. EVs from different stem cells have different mechanisms and targets, but the great potential for wound healing may change the dilemma of amputation risk that diabetic patients face.

Additionally, EVs with adverse effects in DFU are also reported. For instance, EVs produced from HUVECs stimulated by advanced glycation end products are adverse to collagen synthesis by activating the autophagy of human skin fibroblasts, thereby delaying the wound healing process. Further studies have demonstrated that miR-106-5p is highly expressed in EVs secreted by ECs treated with advanced glycation end products and exudate from DFU patients. The expression of extracellular signal-regulated kinase 1/2 is inhibited in fibroblasts after the uptake of miR-106-5p, which inhibits collagen synthesis [132]. A study indicated that exosomes from human-immortalized epidermal cells pretreated with high glucose inhibited HUVEC migration and tube formation, ultimately inhibiting wound healing in diabetic mice. This process may relate to the YY1/HDAC8 singling pathway mediated by LINC01435 in this kind of exosome [133].

**Fig. 4:**
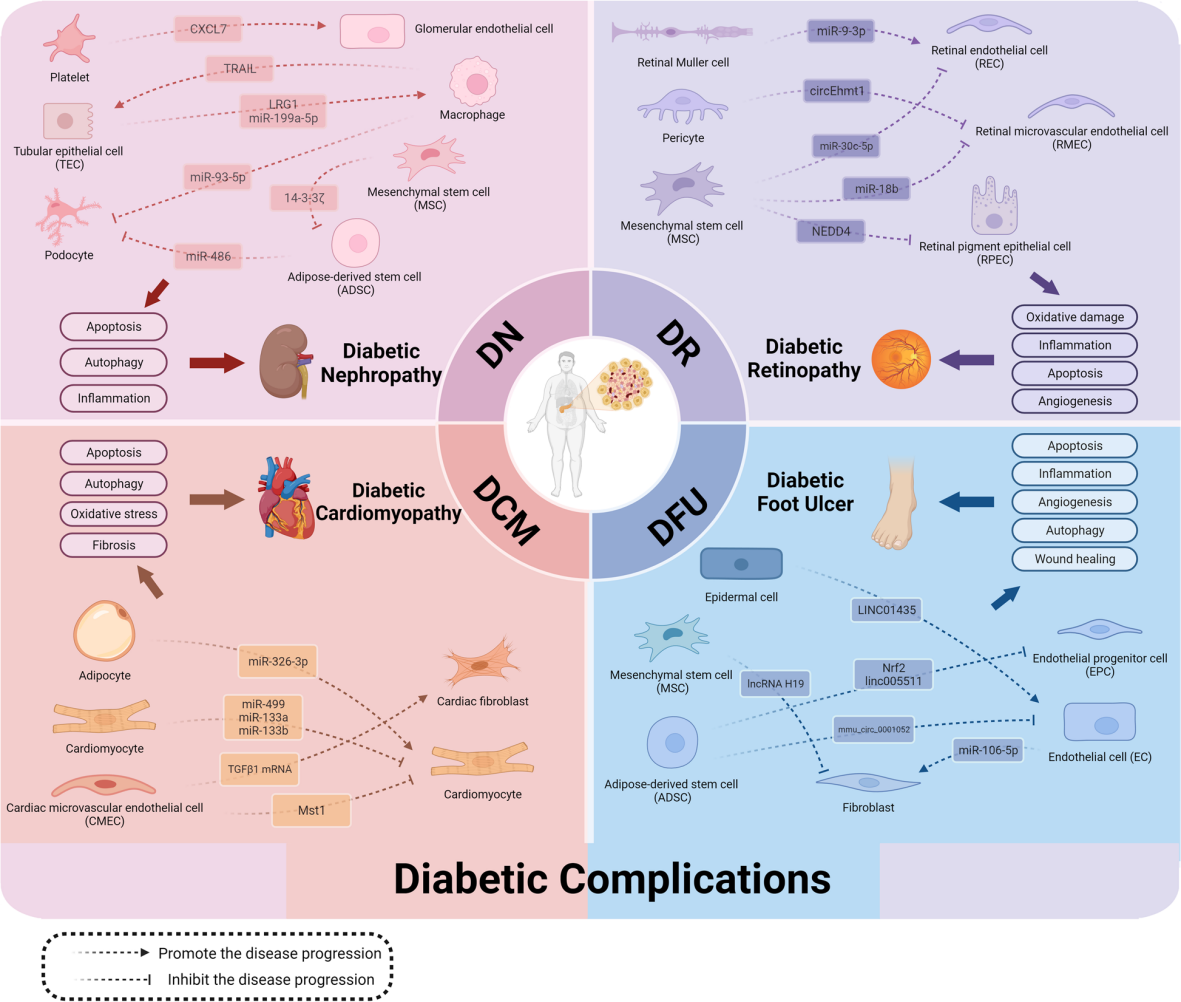
Intercellular communication that EV cargoes mediate regulates diabetic complication progression Cargoes from different donor cells, including tubular epithelial cells (TECs), macrophages, mesenchymal stem cells (MSCs), adipose-derived stem cells (ADSCs), platelets, retinal Muller cells, pericytes, adipocytes, cardiomyocytes, cardiac microvascular endothelial cells (CMECs), and epidermal cells can activate or inhibit the specific targets in recipient cells during the progression of diabetic complications, including diabetic nephropathy (DN), diabetic retinopathy (DR), diabetic cardiomyopathy (DCM), and diabetic foot ulcers (DFU). These processes affect cell apoptosis, autophagy, angiogenesis, and inflammation

Intercellular communication that EVs mediate in diabetic complications is briefly shown in Fig. 4.

### Tumors

#### Hemangiomas

Hemangioma, as a common type of benign tumor in infancy, is associated with hemangioma stem cells (HemSCs) and hemangioma endothelial cells (HemECs) in its progression. Studies on EVs in hemangiomas have mainly concentrated on HemSCs and HemECs as recipient cells. Electroplating miR-187-3p mimics into exosomes produced from human adipose MSCs decreases HemSC viability by acting on the Notch signaling pathway in HemSCs [134]. Furthermore, EVs can address the issue of HemSC drug resistance to propranolol, a first-line therapeutic agent for hemangiomas [135]. Exosomes produced by tumor-associated macrophages lower the sensitivity of HemSCs to propranolol by down-regulating the expression of dickkopf-related protein 2, which relies on miR-27a-3p carried in exosomes [135]. MiR-27a-3p has been identified as a new target for hemangioma treatment, especially propranolol-resistant hemangiomas [135]. Interestingly, EVs can also serve as a bridge between HemSCs and HemECs. Exosomes secreted by HemSCs not only promote HemEC proliferation and angiogenesis but also alleviate HemEC apoptosis and cell cycle arrest [136]. This effect is attributed to miR-196b-5p, which binds to cyclin-dependent kinase inhibitor 1B directly. However, it has not been verified in vivo experiments [136]. Hypoxia is a key factor in tumor development. MiR-210, which is carried by hypoxia-induced HUVEC-derived EVs, accelerates HemEC proliferation and migration [137]. Through software prediction, it was discovered that miR-210 possibly targeted homeobox A9, but the mechanism of this target gene for hemangioma has not been validated experimentally [137]. Further research into EV function and related targets in hemangiomas is required.

#### Hematological Malignancies

Hematological malignancies (mainly including leukemia and lymphoma), a kind of hematologic disease caused by mutations in hematopoietic cells, are also related to angiogenesis in the bone marrow microenvironment [138, 139]. EVs stimulate angiogenesis in leukemia by transporting contents from tumor cells to ECs. A study found that vascular endothelial growth factor (VEGF)-containing exosomes derived from acute myeloid leukemia (AML) promoted HUVEC proliferation and tube formation while also inducing vascular endothelial growth factor receptor (VEGFR) expression in HUVECs [140]. The glycolytic inhibitor 2-DG inhibits the ability of AML-derived exosomes to promote HUVEC angiogenesis in vitro, indicating that vascular remodeling depends on glycolysis [140]. However, this research was conducted in vitro, so it has to be confirmed in vivo. Similarly, exosomes released by AML enhance angiogenesis, and bioinformatics analysis has revealed that exosomal miRNAs play essential roles in AML development, but the relationship between specific miRNAs in exosomes and angiogenesis remains to be further studied [141]. Another study demonstrated the ability of chronic myeloid leukemia (CML)-derived exosomes to trigger angiogenesis in the chicken chorioallantoic membrane assay model [142]. Interestingly, the role of CML-derived exosomes in promoting new angiogenesis is offset by using anti-angiogenesis-gold nanoparticles, which offer a new therapeutic strategy for improving the CML tumor microenvironment and inhibiting angiogenesis [142]. In addition, exosomes secreted by chronic lymphocytic leukemia (CLL) induce migration and angiogenesis by transferring chloride intracellular channel 1 to HUVECs to activate the ITGβ1-MAPK/ERK signaling cascade, which provides a novel target for CLL treatment [143]. In vivo evidence has supported the potential of EVs to stimulate angiogenesis in lymphoma compared with leukemia. A study on diffuse large B cell lymphoma (DLBCL) found that exosomes from DLBCL promoted HUVEC tube formation in a dose-dependent manner in vitro and that angiogenesis increased markedly in NOD/SCID mice with the injection of DLBCL-derived exosomes [144]. The underlying mechanism lacks a specific description. Additionally, anti-tumor drugs can function by interfering with the crosstalk between cells that EVs mediate. Combining omacetaxine and curcumin therapy dramatically reduced VEGF levels in exosomes secreted by Raji cells, according to Zhang et al. [145]. Exosomes containing down-regulated VEGFA markedly inhibited HUVEC proliferation, migration, and angiogenesis concurrently [145]. It further proves the involvement of EVs in angiogenesis in hematological malignancies. Deeply understanding EV roles in angiogenesis in hematological malignancies is conducive to developing and researching anti-angiogenic drugs.

#### Other Solid Tumors

Emerging evidence has shown that EVs play important roles in solid tumors, such as oncogenesis, invasion, metastasis, and drug resistance. The tumor microenvironment (TME), which includes vessels, immune cells, mesenchymal cells, and non-cellular components, is tightly related to tumor progression [146]. EVs perform vital roles as mediators in intercellular communication in the TME, especially during angiogenesis. EVs that shuttle between tumor cells and ECs modulate angiogenesis in tumor development. For instance, miR-25-3p contained in colorectal cancer (CRC)-derived exosomes targets KLF2 and KLF4 in ECs, increasing vascular leakage and promoting angiogenesis. In vivo experiments revealed that miR-25-3p disrupts endothelial barrier integrity and contributes to pre-metastatic niche formation, ultimately promoting CRC metastasis in nude mice. Analyzing clinical samples proves that the high level of exosomal miR-25-3p in circulation is connected with CRC metastasis [147]. The lack of oxygen and nutrition in TME is severe as a result of abnormal angiogenesis, while EVs released by tumors in turn further stimulate EC proliferation and angiogenesis [148]. Glutamine-fructose-6-phosphate aminotransferase 1 in nutrient-deficient bladder cancer-derived EVs drives increased hexosamine biosynthetic pathway flux and O-GlcNAcylation level in ECs, ultimately realizing metabolic reprogramming and promoting angiogenesis [148]. Plentiful factors in TME induce tumor cells to overexpress multiple biomolecules that are favorable for tumor survival. Hypoxia is not the only factor, such as TGF-β. Lysyl oxidase-like 4 (LOXL4) is highly expressed in hepatocellular carcinoma (HCC) [149]. Through the paracrine mechanism of exosomes, it can be transferred to HUVECs, promoting angiogenesis, enhancing invasion, and ultimately exacerbating HCC metastasis [149]. It’s interesting to note that hypoxia does not induce LOXL4 expression, while TGF-β is the regulator of LOXL4 expression in HCC [149]. Notably, the same cargo in EVs shows opposite effects in different tumors. MiR-9 is considered an oncogenic factor in multiple cancers. Compared with the healthy control group, the expression of miR-9 in glioma tissues and patient serum was significantly increased. Further studies found that miR-9 secreted from glioma cells was absorbed by HUVECs through exosomes and ultimately promoted angiogenesis. Studies have shown that miR-9 can target collagen type XVIII alpha 1 chain, thrombospondin 2, patched 1, and prolyl hydroxylase domain 3 in ECs, affecting the VEGF signaling pathway [150]. However, in a study by Lu et al., exosomes from nasopharyngeal carcinoma were taken up by ECs, where miR-9 reversed MDK-induced cell migration and tube formation, playing anti-angiogenic roles [151]. Possibly, the downstream signaling pathway and the specific underlying mechanism are different.

Other than invasion and metastasis, EVs have momentous effects on solid tumor resistance through intercellular communication. One study demonstrated that exosomes carrying miR-522 secreted by cancer-associated fibroblasts (CAFs) inhibited arachidonate lipoxygenase 15 expression in gastric cancer cells and ultimately repressed ferroptosis, which is related to gastric cancer chemical resistance [152]. Another report confirmed that bevacizumab, an anti-angiogenesis drug, was detected on the surface of EVs derived from glioblastoma cells after bevacizumab treatment. This is likely a mechanism by which cancer cells protect themselves from drug toxicity [153]. Further research indicated that inhibiting EV secretion with GW4869 enhanced the therapeutic effect of bevacizumab on glioblastoma cells and reduced cell viability, but the specific mechanism remains to be elucidated [153]. Additionally, due to the heterogeneity of tumors, wild epidermal growth factor receptor (EGFR) non-small cell lung cancer (NSCLC) promotes the resistance of mutated EGFR NSCLC to osimertinib by transferring wild EGFR to mutant NSCLC [154].

**Fig. 5:**
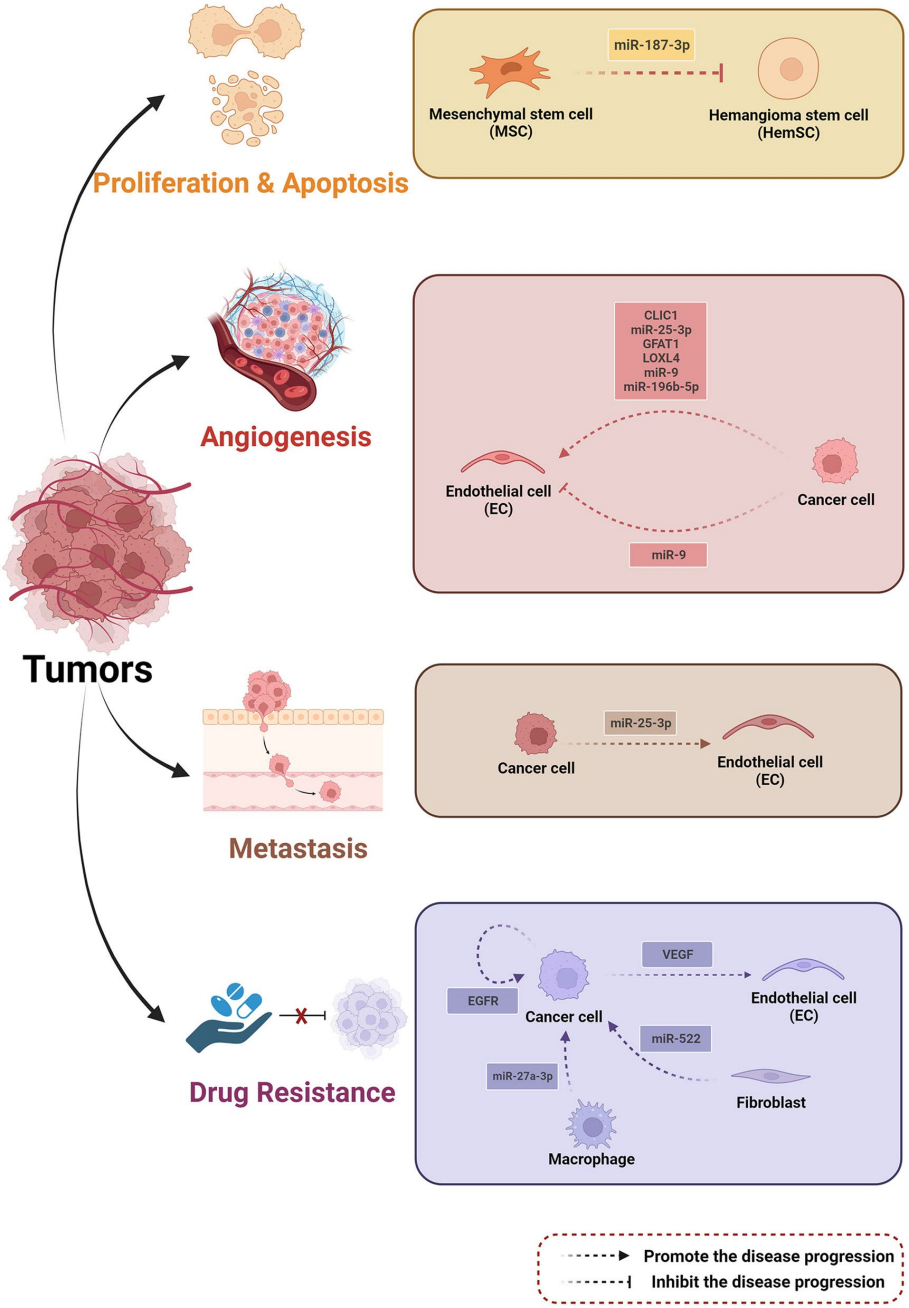
Intercellular communication that EV cargoes mediate regulates the tumor progression As tumors such as hemangiomas, hematological malignancies, and other solid tumors advance, cargoes from various donor cells, including mesenchymal stem cells (MSCs), cancer cells, macrophages, and fibroblasts can activate or inhibit specific targets in recipient cells. These processes affect tumor development by regulating cell proliferation, apoptosis, angiogenesis, metastasis, and drug resistance

Figure 5 provides a brief illustration of intercellular communication that EVs mediate in tumors.

### Other Vascular Diseases

#### Acute Lung Injury (ALI)

The influence of EVs on alveolar macrophages, neutrophils, and pulmonary microvascular endothelial cells (PMVECs) is closely associated with the progression of ALI, particularly in inflammation response and blood-air barrier integrity. For instance, exosomal miR-30d-5p from polymorphonuclear neutrophils was internalized by macrophages and then targeted Sirt1 and suppressor of cytokine signaling 1, which ultimately activated the NF-κB signaling pathway and induced macrophage M1 polarization and pyroptosis, aggravating sepsis-related ALI [155]. In contrast, adipose-derived MSC-derived exosomes transfer mtDNA to alveolar macrophages to compensate for damaged mitochondria, thereby enhancing macrophage metabolism and improving immune homeostasis, eventually reducing inflammation and improving ALI [156]. Similarly, a recent study confirmed that miR-125b-5p in exosomes from ADSCs reduced inflammation-stimulated PMVEC ferroptosis through the Keap1/Nrf2/GPX4 axis, hence alleviating ALI [157]. Furthermore, Zhou et al. highlighted that exosomes from EPCs administrated intratracheally reduced alveolar edema and pulmonary inflammatory cell infiltration in ALI mice. Further research revealed that miR-126-3p and miR-126-5p notably increased the expression of tight junction proteins (Claudin1, Claudin4, and occludin) in human small airway epithelial cells and improved the endothelium barrier integrity in vitro [158, 159].

Collectively, cell–cell interactions that EVs mediate show great potential for ALI therapies since this process provides substantial therapeutic targets.

#### Acute Kidney Injury (AKI)

Emerging evidence has indicated that EVs play a crucial role in renal protection and regeneration in AKI, which involves TECs, MSCs, immune cells, and other cells. Cao et al. provided evidence that MSC-EVs specifically accumulated in kidney injury areas and transported miR-200a-3p to proximal TECs [160]. Subsequently, miR-200a-3p activated the Keap1-Nrf2 signaling pathway to enhance proximal TEC mitochondrial function, ultimately improving renal function during AKI progression [160]. Similarly, EVs produced by MSCs were found to mitigate mitochondrial dysfunction and the inflammatory response by transporting mitochondrial transcription factor A and mtDNA, ultimately ameliorating renal damage [161]. Additionally, sEVs secreted by hypoxic proximal TECs stimulated capillary endothelial proliferation around renal tubules and angiogenesis after AKI [162]. VEGFA found in this kind of sEV plays a pivotal role in peritubular capillary repair and renal regeneration [162]. In brief, intercellular communications that EVs mediate function critically in AKI, and studies about specific mechanisms are ongoing.

## Clinical Application Potential of EVs in Vascular Diseases

### Therapeutic Targets

Researchers have identified numerous potential therapeutic targets by studying the intercellular communication that EVs mediate in vascular diseases, including cargos (miRNAs, circRNAs, lncRNAs, proteins, etc.) in EVs and genes in target cells, which are discussed in the second part and summarized in Table 1. Interfering with cargo identification in EVs and target genes in recipient cells or designing and delivering content mimics of EVs, are promising treatment strategies for vascular diseases.

**Table 1:** Preclinical studies assessing the effect of intercellular communications that EVs mediate in vascular diseases

Disease	Cargo	Target/Pathway	Donor Cell	Recipient Cell	Main Function	Reference
**Ischemic Stroke (IS)**	MiR-98	Platelet-activating factor receptor	Neuron	Microglia	Attenuate neuronal death after IS	[39]
	Transmembrane tumor necrosis factor	-	Regenerative microglia	Oligodendrocyte precursor cell (OPC)	Promote the OPC maturation and ameliorate neurological function after IS	[40]
	MiR-23a-5p	OLIG3-oligodendrocyte transcription factor 3	M2 microglia	OPC	Promote white matter repair and functional recovery after IS	[41]
	MiR-124	Signal transducer and activator of transcription 3	M2 microglial	Astrocyte	Attenuate glial scar formation and promote recovery after IS	[42]
	-	-	Astrocyte	Neuron	Promote axonal growth and neuronal survival after IS	[44]
	-	-	Activated astrocyte	Neuron	Regulate axonal growth after ischemia	[45]
	MiR-25-3p	p53 pathway	Mesenchymal stem cell (MSC)	Neuron	Inhibit autophagy and have neuroprotective effects	[46]
	MiR-125b-5p	Toll-like receptor 4 (TLR4) and nuclear transcription factor-kappaB (NF-κB) signaling	MSC	Astrocyte	Attenuate BBB integrity disruption induced by tPA and alleviate hemorrhage	[47]
	TGF-β, Smad2, and Smad4	TGF-β/Smad signaling pathway	Embryonic stem cell (ESC)	Regulatory T cell	Alleviate neuroinflammation and promote long-term neurological recovery after IS	[48]
**Intracerebral Hemorrhage (ICH)**	-	-	Multipotent mesenchymal stromal cell	-	Promote neurogenesis and angiogenesis and improve functional recovery after ICH	[50]
	MiR-23b	Phosphatase and tensin homolog deleted on chromosome 10 (PTEN) and PTEN/Nrf2 pathway	Bone marrow mesenchymal stem cell (BMSC)	Microglia/macrophage	Exhibit antioxidant effects and promote neurologic function recovery after ICH	[51]
	MiR-19b-3p	Iron regulatory protein 2	Adipose-derived stem cell (ADSC)	Neuron	Alleviate neuronal ferroptosis and improve neurological function after ICH	[52]
	Signal regulatory protein α (SIRPα)- variants	CD47	MSC	Red blood cell	Accelerate hematoma clearance and ameliorate white matter injury after ICH	[53]
	MiR-383-3p	Activating transcription factor 4	Activated microglia	Neuron	Promote neuron necroptosis after ICH	[54]
**Vascular Dementia (VD)**	MiR-154-5p	Protein kinase AMP-activated catalytic subunit alpha 2 (PRKAA2)	-	Endothelial progenitor cell (EPC)	Inhibit EPC functions and inhibit angiogenesis	[58]
	MiR-132-3p	Ribosomal arginine synthetase 1 and Ras/Akt/GSK-3β pathway	MSC	Neuron	Improve synaptic dysfunction and cognitive decline in VD	[59]
	MiR-17-5p, miR-18a-5p, miR-21-5p, miR-29a-3p, and let-7a-5p	Rapamycin complex 1	ESC	Hippocampal neural stem cell (HNSC)	Ameliorate senescence-related neurogenesis dysfunction and cognitive impairment in VD	[61]
	MIAT	MiR-34b-5p/CALB1 axis	HNSC	Hippocampal neuronal cell	Improve cognitive disorders in VD	[62]
**Cerebral Small Vascular Disease (CSVD)**	-	-	MSC	Microglia	Attenuate inflammation in CSVD	[64]
**Hypertension**	MiR-320d/423-5p	-	Endothelial cell (EC)	Subendothelial smooth muscle cell (SMC)	Cause arterial remodeling in hypertension	[70]
	MiR-483-3p	Angiotensin-converting enzyme I (ACE I), CTGF, TGF-β	EC	SMC	Inhibit endothelial dysfunction and exert protective effects on EC function during the onset of hypertension	[71]
	ACE	-	Adventitial fibroblast	Vascular smooth muscle cell (VSMC)	Cause VSMC proliferation and vascular remodeling and hypertension	[72]
	MiR-155-5p	ACE	Adventitial fibroblast	VSMC	Attenuate VSMC proliferation and vascular remodeling in hypertension	[73]
	MiR-135a-5p	Fibronectin type III domain containing 5 (FNDC5)	Adventitial fibroblast	VSMC	Promote VSMC proliferation in hypertension	[74]
**Atherosclerosis**	MiR-92a	Krüppel-like factor 4 (KLF4)	EC	Macrophage	Cause atherosclerotic lesion formation in atherosclerosis	[78]
	-	-	Monocyte/platelet	EC	Trigger differential activation of human atherosclerotic plaque in atherosclerosis	[79]
	MiR-221	N-acetyltransferase-1	BMSC	SMC	Inhibit atherosclerotic plaque formation against atherosclerosis	[80]
	MiR-146a	Src signal pathway	MSC	Senescent EC	Mitigate EC senescence and stimulate angiogenesis during atherosclerosis	[81]
	MiR-27b-3p	Peroxisome proliferator-activated receptor α	Visceral adipocyte	EC	Promote endothelial inflammation and facilitate atherogenesis in atherosclerosis	[82]
	MiR-155	BCL2, MCL1, TIMP3, BCL6	Nicotine-stimulated monocyte	EC	Promote plaque formation and trigger vascular endothelial injury in atherosclerosis	[83]
	MiR-21-3p	PTEN	Nicotine-stimulated macrophage	VSMC	Increase VSMC migration and proliferation and accelerate atherosclerosis development	[84]
**Acute Mycardial Infarction (AMI)**	-	C-X-C chemokine receptor type 4	Infarcted cardiomyocyte	Bone marrow monocyte	Promote cardiac repair after AMI	[86]
	CircUbe3a	MiR-138-5p/RhoC axis	M2 macrophage	Cardiac fibroblast	Exacerbate myocardial fibrosis after AMI	[87]
	MiR-155	RAC1, RAK2, Sirt1, Enos, and AMPKa2	M1 macrophages	EC	Inhibit angiogenesis and aggravate heart injury after AMI	[88]
	MiR-125b-5p	-	MSC	Cardiomyocyte	Inhibit autophagic after AMI	[90]
	MiR-486-5p	Matrix metalloproteinase 19	Hypoxia-preconditioned MSC	Cardiac fibroblast	Promote angiogenesis after AMI	[91]
	-	Foxo3	-	Regulatory T cell	Inhibit cardiac inflammation and promote cardiac repair after AMI	[92]
**Myocardial Ischemia–Reperfusion Injury (MIRI)**	Linc-ROR	MiR-145-5p	Cardiomyocyte	Cardiac microvascular endothelial cell (CMEC)	Increase the survival of CMECs and CMs and attenuate MIRI injury	[95]
	Cardioprotective proteins	-	EC	Cardiomyocyte	Attenuate cardiac injury after MIRI	[96]
	MiR-24-3p	CCR2	KLF2-overexpressing EC	Monocyte	Inhibit monocyte recruitment and attenuate MIRI	[97]
	MiR-130b-3p	AMPKα1/α2, Birc6, and Ucp3	Diabetic adipocytes	Cardiomyocyte	Induce proapoptotic/cardiac harmful effects and exacerbate MIRI	[98]
	Damaged mitochondria	-	Energetically stressed adipocyte	Cardiomyocyte	Protect cardiomyocytes against oxidative stress after MIRI	[99]
	MiR-210	Ephrin A, protein tyrosine phosphatase 1B, and death-associated protein kinase 1	ADSC	EC and cardiomyocyte	Promote angiogenesis and inhibit apoptosis after MIRI	[100]
**Heart Failure (HF)**	MiR-378	Mitogen-activated protein kinase kinase 6	Cardiomyocytes under the overloaded condition	Cardiac fibroblasts	Inhibit excessive fibrosis in HF	[102]
	MiR-21-5p	PTEN/Akt signaling pathway	Cardiac stromal cell	Cardiomyocyte	Enhance angiogenesis and cardiomyocyte survival in HF	[103]
	-	MiR-200b	Human trophoblast stem cell	-	Have antiapoptotic and anti-inflammatory effects and attenuate doxorubicin-induced cardiac injury in HF	[105]
	MiR-100-5p	NADPH oxidase 4	MSC	Cardiomyocyte	Suppress oxidative stress and play cardioprotective roles in HF	[101]
	-	-	ESC	Macrophage	Attenuate pyroptosis and cardiac remodeling induced by Doxorubicin	[106]
	Mitochondrial transcription factor A and mtDNA	-	MSC	-	Mitigate mitochondrial injury and inflammation in AKI	[161]
	VEGF-A	Vascular endothelial growth factor receptor (VEGFR)	Tubular epithelial cell (TEC)	-	Promote angiogenesis after AKI	[162]
**Diabetic Nephropathy (DN)**	Chemokine ligand 7	Rapamycin complex 1 pathway	platelet	Glomerular endothelial cell	Exacerbate glomerular endothelial damage and promote the progression of DN	[107]
	MiR-19b-3p	Suppressor of cytokine signaling 1	TEC	Macrophage	Drive the development of tubulointerstitial inflammation in DN	[108]
	Leucine-rich α-2-glycoprotein 1	TGF-β R1	TEC	Macrophage	Promote inflammation and induce macrophages to secret EVs in DN	[109]
	Tumor necrosis factor-related apoptosis-inducing ligand (TRAIL)	Death receptor 5 (DR5)	Macrophage	TEC	Induce TEC apoptosis and promote DN	[109]
	MiR-199a-5p	Klotho	TEC	Macrophage	Stimulate M1 polarization and accelerate DN	[110]
	MiR-93-5p	TLR4	Macrophage	Podocyte	Alleviate podocyte apoptosis and inhibit DN development	[111]
	-	mTOR	MSC	-	Suppress autophagy and alleviate DN	[112]
	MiR-486	Smad1/mTOR signaling pathway	ADSC	Podocyte	Promote autophagy flux, inhibit apoptosis, and ameliorate DN	[113]
	14–3-3 ζ	Yes-associated protein	MSC	Podocyte	Promote autophagy and prevent DN	[114]
**Diabetic Retinopathy (DR)**	MiR-9-3p	Sphingosine-1-phosphate receptor 1	Retinal Muller cell	Retinal endothelial cell (REC)	Promote abnormal angiogenesis and aggravate DR	[115]
	CircEhmt1	NFIA/NLRP3 pathway	Pericyte	Retinal microvascular endothelial cell (RMEC)	Improve microvascular dysfunction and ameliorate DR	[116]
	MiR-30c-5p	Phospholipase C gamma 1	MSC	REC	Inhibit inflammation in DR	[117]
	Neuronal precursor cell-expressed developmentally downregulated 4	PTEN	MSC	Retinal pigment epithelium cell	Inhibit apoptosis and oxidative damage and prevent DR development	[118]
	MiR-192	Integrin alpha 1	MSC	-	Alleviate inflammation and angiogenesis in DR	[119]
	MiR-18b	Mitogen-activated protein kinase 1	MSC	RMEC	Inhibit inflammation and apoptotic in DR	[120]
**Diabetic Cardiomyopathy (DCM)**	MiR-326-3p	Rictor	Senescent adipocyte	Cardiomyocyte	Cause mitochondrial dysfunction and cardiac diastolic dysfunction in DCM	[123]
	Mst1	Daxx	CMEC	Cardiomyocyte	Inhibit autophagy, promote apoptosis, and alleviate DCM	[124]
	MiR-499, miR-133a, and miR-133b	-	Cardiomyocyte	Cardiomyocyte	Inhibit autophagy and oxidative stress and attenuate DCM	[125]
	-	-	Cardiac parasympathetic neuron	Cardiac fibroblast	Inhibit apoptosis and DCM progression	[126]
	TGF-β1 mRNA	-	CMEC	Cardiac fibroblast	Promote myocardial fibrosis in DCM	[127]
**Diabetic Foot Ulcers (DFU)**	Nuclear factor-erythroid factor 2 (Nrf2)	-	ADSC	EPC	Promote wound healing in DFU	[128]
	Linc00511	Progestin and adipoQ receptor 3	ADSC	EPC	Promote angiogenesis and attenuate DFU	[129]
	Mmu_circ_0001052	MiR-106a-5p	ADSC	EC	Promote angiogenesis and wound healing and attenuate DFU	[130]
	LncRNA H19	MiR-152-3p	MSC	Fibroblast	Inhibit apoptosis and inflammation and ameliorate wound ulcers in DFU	[131]
	MiR-106-5p	Extracellular signal-regulated kinase 1/2	EC	Fibroblast	Activate autophagy and inhibit wound healing in DFU	[132]
	LINC01435	YY1	Epidermal cell	EC	Delay the wound healing process of DFU	[133]
**Hemangioma**	MiR-187-3p	Notch signaling	MSC	Hemangioma stem cell (HemSC)	Suppress HemSC proliferation in hemangioma	[134]
	MiR-27a-3p	Dickkopf-related protein 2	Tumor-associated macrophage	HemSC	Suppress propranolol sensitivity and decrease apoptosis in hemangioma	[135]
	MiR-196b-5p	Cyclin-dependent kinase inhibitor 1B	HemSC	Hemangioma endothelial cell (HemEC)	Enhance proliferation and angiogenesis and attenuate apoptosis of HemECs in hemangioma	[136]
	MiR-210	Homeobox A9	Hypoxia-induced EC	HemEC	Enhance the proliferation and migration of HemECs and inhibit apoptosis of HemECs in hemangioma	[137]
**Hematological Malignancies**	Vascular endothelial growth factor (VEGF) and vascular endothelial growth factor receptor (VEGFR) messenger RNA	VEGFR	Acute myeloid leukemia (AML) cell	EC	Stimulate vascular remodeling and chemoresistance in AML	[140]
	-	-	AML cell	EC	Promote angiogenesis in AML	[141]
	-	-	Chronic myeloid leukemia (CML) cell	EC	Promote angiogenesis in CML	[142]
	Chloride intracellular channel 1	ITGβ1	Chronic lymphocytic leukemia (CLL) cell	EC	Promote angiogenesis in CLL	[143]
	-	-	Diffuse large B cell lymphoma (DLBCL) cell	EC	Promote angiogenesis in DLBCL	[144]
	VEGF	VEGF/Akt signaling pathway	Lymphoma cells treated with curcumin and omacetaxine	EC	Inhibit angiogenesis in lymphoma	[145]
**Other Solid Tumors**	miR-25-3p	KLF2 and KLF4	CRC cell	EC	Promote angiogenesis and metastasis in CRC	[147]
	Glutamine-fructose-6-phosphate aminotransferase 1	-	Bladder cancer cell	EC	Realize metabolic reprogramming and promote angiogenesis in bladder cancer	[148]
	Lysyl oxidase-like 4 (LOXL4)	The FAK/Src pathway	Hepatocellular carcinoma (HCC)	EC	Promote angiogenesis, enhance invasion, and exacerbate HCC metastasis	[149]
	MiR-9	Collagen type XVIII alpha 1 chain, thrombospondin 2, patched 1, and prolyl hydroxylase domain 3	Glioma cell	EC	Promote angiogenesis in glioma	[150]
	MiR-9	MDK	Nasopharyngeal carcinoma cell	EC	Inhibit angiogenesis in nasopharyngeal carcinoma	[151]
	MiR-522	Arachidonate lipoxygenase 15	Cancer-associated fibroblast (CAF)	Gastric cancer cell	Inhibit ferroptosis and induce chemical resistance	[152]
	EGFR	PI3K/AKT and MAPK pathways	Wild EGFR non-small cell lung cancer (NSCLC) cell	Mutated EGFR NSCLC cell	Promote the resistance to osimertinib	[154]
**Acute Lung Injury (ALI)**	MiR-30d-5p	Suppressor of cytokine signaling 1 and Sirt1	Polymorphonuclear neutrophil	Macrophage	Induce M1 macrophage polarization and pyroptosis and aggravate ALI	[155]
	mtDNA	-	MSC	Macrophage	Reduce inflammation and improve ALI	[156]
	MiR-125b-5p	Keap1	ADSC	Pulmonary microvascular endothelial cell (PMVEC)	Alleviate inflammation and improve ALI	[157]
	MiR-126-3p and MiR-126-5p	Phosphoinositide-3-kinase regulatory subunit 2	EPC	EC	Inhibit inflammation and microvascular dysfunction and alleviate ALI	[158, 159]
**Acute Kidney Injury (AKI)**	MiR-200a-3p	Keap1	MSC	TEC	Enhance mitochondrial function and improve renal function during AKI	[160]
	Mitochondrial transcription factor A and mtDNA	-	MSC	-	Mitigate mitochondrial injury and inflammation in AKI	[161]
	VEGFA	VEGFR	TEC	-	Promote angiogenesis after AKI	[162]

Additionally, investigating the process of EV biogenesis, secretion, and uptake can shed light on prospective therapeutic targets for vascular diseases, mainly by modulating the intercellular crosstalk that EVs mediate. Blocking or promoting these EV processes may play an effective role in treating vascular diseases. For example, Rab27a and Rab27b play important roles in exosome release from alveolar macrophages stimulated by lipopolysaccharides, which aggravates ALI. However, IL-25 secreted by alveolar epithelial cells inhibits Rab27a and Rab27b expression in macrophages, ultimately inhibiting exosome release and reducing TNF-α level, hence providing a new therapeutic strategy for ALI [163]. Everolimus, a rapamycin derivative, stimulates NSCLC cells to secrete exosomes loaded with miR-7-5p, thereby reducing its antitumor activity. The knockdown of Rab27A or Rab27B inhibits the exocytosis of exosomes carrying miR-7-5p and attenuates the resistance of NSCLC cells to everolimus [164].

**Fig. 6:**
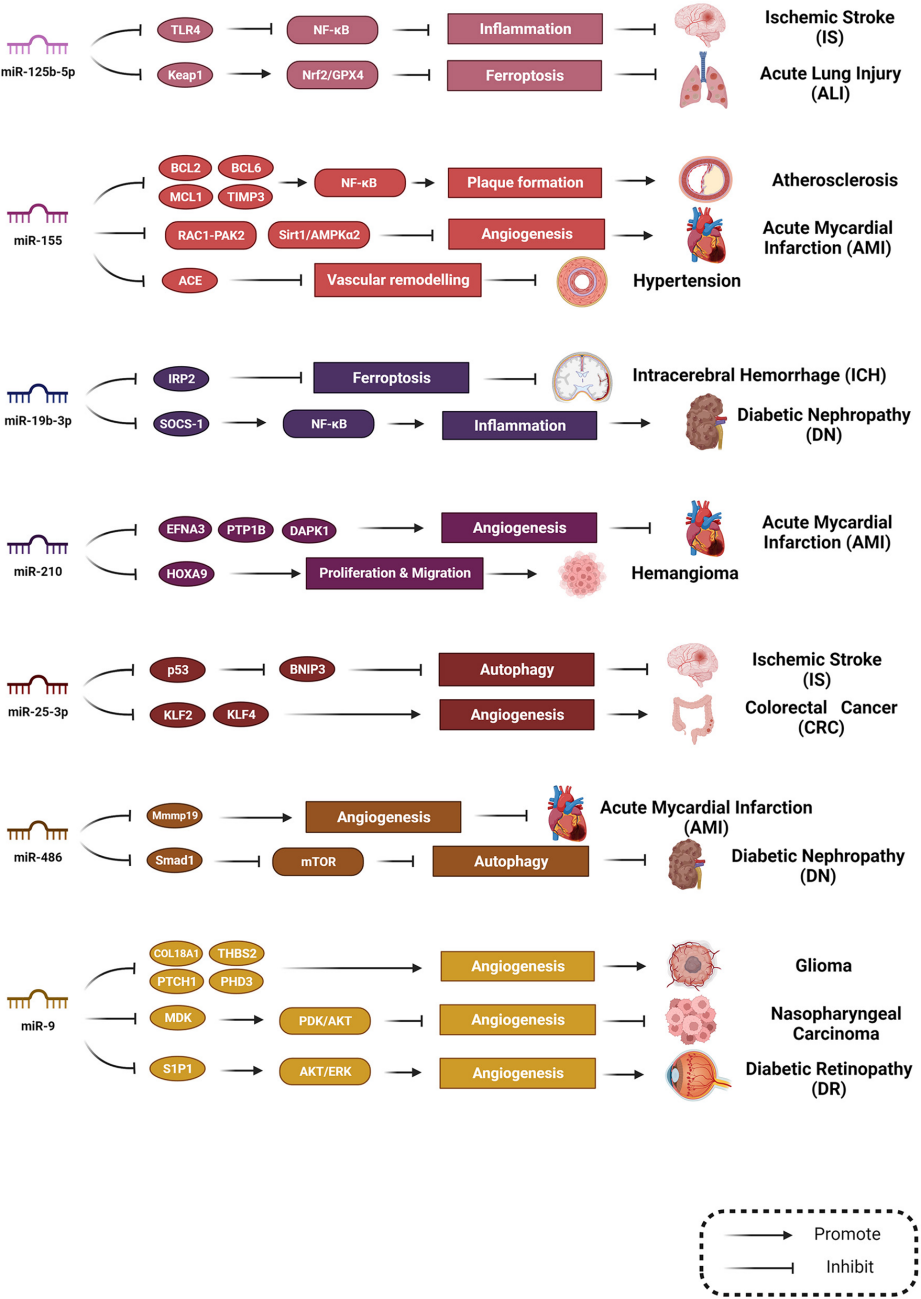
The same cargo has similar, different, or opposite effects through diverse mechanisms in vascular diseases MiR-125b-5p, miR-155, miR-19b-3p, miR-210, miR-25-3p, miR-486, and miR-9 have different downstream targets that promote or inhibit diverse physiological and pathological processes in different types of vascular diseases. The effects of the same cargo can be similar, different, or even opposite

GW4869, an EV-release inhibitor, is commonly used in the scientific community. In a study of MIRI, Ge et al. found that reperfusion induced an increase in cardiac EV release, triggering an inflammatory response that exacerbated heart injury, whereas GW4869 treatment inhibited EV release while reducing cardiac injury [165]. Similarly, Patwardhan et al. used GW4869 to block exosome secretion and attenuate extracellular matrix stiffness, thereby modulating the motility and contractility of breast cancer cells and inhibiting breast cancer invasion [166]. However, the problem of directly blocking EV release should not be overlooked. The same cargo performs similar, different, and even opposite functions through diverse mechanisms in different vascular diseases and the effects of EVs from different sources may be similar or opposite, as shown in Fig. 6. The same miRNAs can interact with different targets and functions in various vascular diseases. Mechanisms that EVs mediate may be related to the nature of their targets. Completely blocking or promoting EV release is not appropriate for clinical applications because of various cargo roles. This should also be considered. Otherwise, it may lead to other pathological processes in the body. A comprehensive examination of the various roles of EVs and their cargo is a challenge requiring further study.

### Diagnostic and Prognostic Biomarkers

**Fig. 7:**
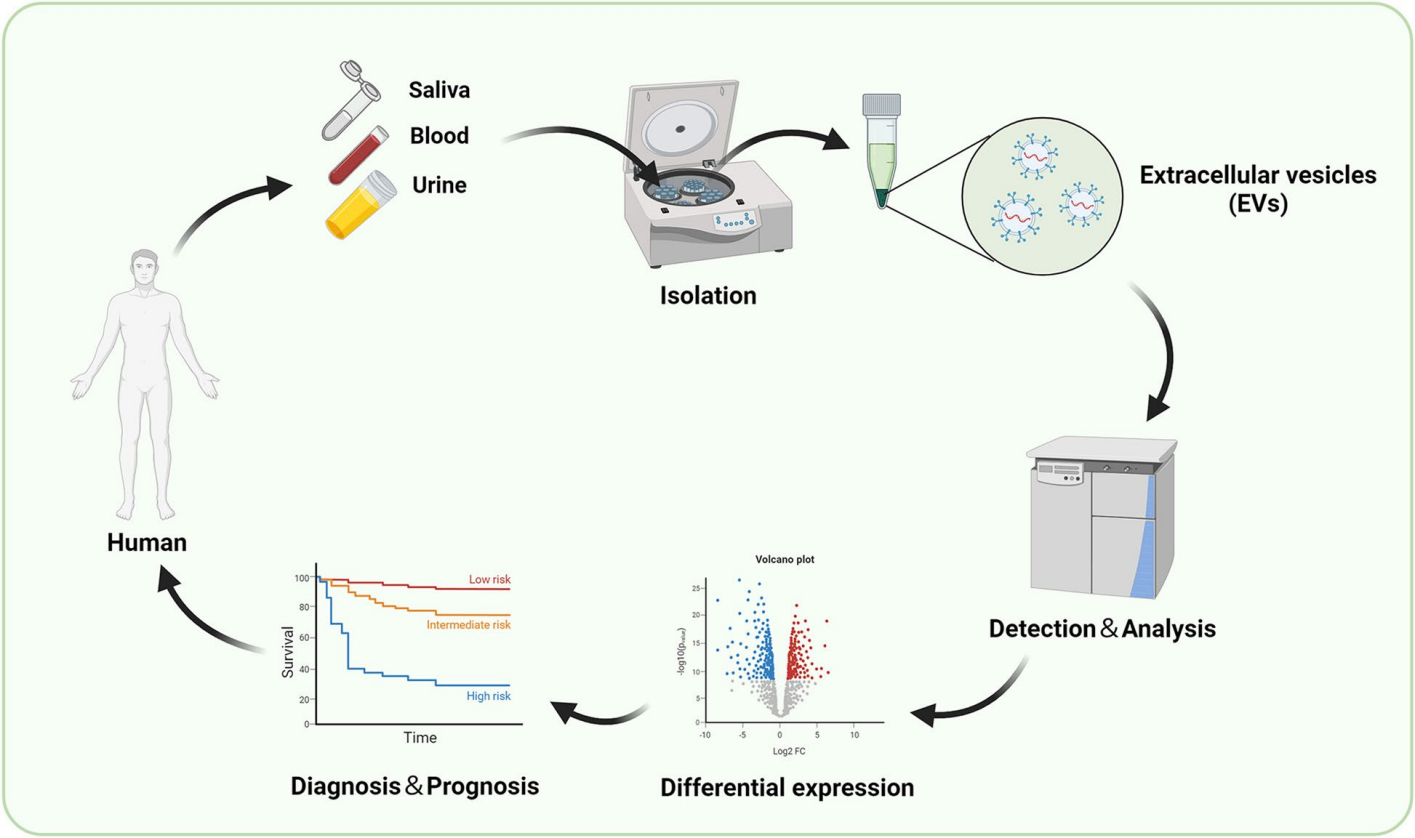
The procedure of EV cargoes as non-invasive biomarkers for diagnosis and prognosis First, blood, urine, saliva, and other samples are obtained from humans. Second, extracellular vesicles (EVs) are isolated from these samples. Third, differential expression in EVs is analyzed using advanced instruments or sequencing techniques, which is ultimately favorable for analyzing the correlation between differential expression and vascular disease progression to identify noninvasive biomarkers for diagnosis and prognosis

EV cargoes from blood and urine are gradually being used as noninvasive biomarkers for vascular disease diagnosis and prognosis, as EVs exist in body fluids and exhibit different content variations between healthy people and patients (Fig. 7). Most studies on diagnostic biomarkers focus on noncoding RNAs and proteins in EVs, which aim to understand the different stages and prognoses of vascular disease and distinguish between similar diseases (Table 2).

**Table 2:** Cargoes carried in EVs as diagnostic and prognostic biomarkers from clinical samples in vascular diseases

Disease	EV provenance	Cargo	Expression	Clinical significance	Reference
**Ovarian Cancer**	Human malignant ascites and plasma	MiR-1246, miR-1290, miR-483, miR-429, miR-34b-3p, miR-34c-5p, miR-145-5p, miR-449a	MiR-1246, miR-1290, miR-483, miR-429 upregulated and miR-34b-3p, miR-34c-5p, miR-145-5p, miR-449a downregulated	Serve as a miRNA-based diagnostic signature to judge the metastasis in ovarian cancer	[167]
**Ischemic Stroke (IS)**	Human plasma	Circ_0043837 and circ_ 0001801	Both downregulated	Serve as two-circRNA-based risk factors to predict and diagnose large artery atherosclerotic stroke and plaque rupture	[168]
**Ischemic Stroke (IS)**	Human serum	MiR-124-3p	Downregulated	Serve as a predictive and diagnostic biomarker for the early stage of IS	[169]
**Ischemic Stroke (IS)**	Human plasma	MiR-21-5p and miR-30a-5p	MiR-21-5p upregulated in SIS and RIS, miR-30a-5p upregulated in HIS and downregulated in AIS	Serve in combination to distinguish and diagnose different phases of IS	[170]
**Cerebral Small Vessel Disease (CSVD)**	Human plasma	MiR-223-3p	Upregulated	Serve as a predictive biomarker for CSVD	[171]
**Cerebral Small Vessel Disease (CSVD)**	Human plasma	MiR-320e	Downregulated	Serve as a potential biomarker for CSVD	[172]
**Bladder Cancer**	Human urine	Ephrin type-A receptor 2 (EphA2)	Upregulated	Serve as a diagnostic biomarker for early bladder cancer	[173]
**Breast Cancer**	Human plasma	EphA2	Upregulated	Serve as a prognosis biomarker related to metastasis in breast cancer	[174]
**Lung Cancer**	Human serum	MiR-7	Downregulated	Serve as a prognosis biomarker related to gefitinib sensitivity in lung cancer	[175]
**Aortic Stenosis**	Human plasma	CD172a	Upregulated	Serve as a prognosis biomarker for aortic stenosis	[176]
**Stroke**	Human plasma	MiRNAs	-	Serve as biomarkers to distinguish IS and ICH	[177]
**Alzheimer's Disease (AD) and Vascular Dementia (VD)**	Human serum	MiR-22*, miR-23a, miR-29a, miR-34b, miR-125b, and miR-130b	MiR-22*, miR-23a, miR-29a, miR-34b, miR-125b, and miR-130b upregulated in AD patients while downregulated in VD patients	Serve as biomarkers to diagnose and discriminate AD and VD	[178]

MiRNA^*^ is a special kind of miRNAs. In the process of miRNA participating in the regulation of target genes, the miRNA chain that does not enter RISC is called passenger and is crowned with an asterisk (^*^), which has lower stability and is usually degraded.

EVs can be used to diagnose disease progression and provide a basis for determining the vascular disease stage. For example, Wang et al. found that eight miRNAs (miR-1246, miR-1290, miR-483-5p, miR-429, miR-34b-3p, miR-34c-5p, miR-449a, and miR-145-5p) performed well in discrimination by analyzing the miRNA expression profiles in the ascites and plasma of patients with ovarian cancer or benign gynecological diseases. They were then used to establish an ovarian cancer EV miRNA (OCEM) signature. Further analysis suggested that the OCEM signature clearly distinguished benign peritoneal fluids and malignant ascites, as well as benign and malignant plasma, indicating that it can be used to diagnose ovarian cancer in the metastatic stage [167]. By studying circRNA expression in serum exosomes of patients with acute IS, researchers found that exosomal circ_0043837 and circ_ 0001801 can be used as predictive biomarkers for large artery atherosclerotic stroke, are superior to plasma circRNAs, and provide potential diagnostic markers for the early onset and prevention of IS [168]. Similarly, exosomal miR-124-3p is downregulated in the serum of patients with acute cerebral ischemia and is a promising diagnostic biomarker for early-stage IS [169]. Interestingly, a combination of exosomal miR-21-5p and miR-30a-5p in plasma are promising diagnostic biomarkers for distinguishing different IS stages, including hyperacute phase IS (HIS, within 6 h), acute phase IS (AIS, including days 1–3 and days 4–7), subacute phase IS (SIS, days 8–14), and recovery phase IS (RIS, days > 14). The miR-21-5p level in SIS and RIS was notably higher than that in nonstroke controls, and miR-30a-5p expression in HIS was higher, whereas that in AIS (days 1–3) was lower than that in nonstroke controls [170]. Additionally, the exosomal miR-223-3p levels in the plasma of CSVD patients positively correlated with the plasma concentrations of homocysteine and C-reactive protein. This may serve as a predictive diagnostic marker for CSVD and cognitive dysfunction [171]. Gao et al*.* demonstrated that miR-320e from the plasma exosomes of patients with CSVD was also a potential predictive biomarker for CSVD [172]. In addition to blood, EVs derived from urine in bladder cancer serve as diagnostic markers. One study found that ephrin type-A receptor 2 (EphA2) was prominently upregulated in urinary EVs, and EV-EphA2-CD9 ELISA performed well in diagnosing early bladder cancer [173]. EV cargoes are sensitive predictive markers that contribute to the prevention of vascular disease progression.

Additionally, EVs are associated with the prognosis of vascular diseases. A breast cancer study demonstrated that EphA2 in circulating exosomes indicates poor prognosis and metastasis [174]. Plasma EphA2 level was positively correlated with poor prognosis in patients with metastatic breast and was markedly higher than that in patients with nonmetastatic breast cancer [174]. Another study showed that healthy controls have drastically higher miR-7 levels in their serum than patients with lung cancer, and that high miR-7 levels in patient serum are associated with high sensitivity to gefitinib and longer survival [175]. Interestingly, Anselmo et al. demonstrated that the higher the levels of circulating cardiomyocyte-derived CD172a^+^ EVs in patients with aortic stenosis, the better the prognosis after transcatheter aortic valve replacement [176].

MiRNAs play a non-negligible role in distinguishing similar vascular diseases. Kalani et al. analyzed the difference in plasma miRNA expression between patients with ICH and those with IS [177]. They further used 25 miRNA classifiers to analyze the differences and found that their accuracy in distinguishing hemorrhagic from IS was 0.813 ± 0.003 [177]. However, changes in specific miRNA expression levels require further study [177]. This study will help medical practitioners quickly identify patients with cerebral ischemia or exclude cerebral ischemia simultaneously, thereby contributing to rapid diagnosis and treatment [177]. Additionally, miRNAs from exosomes can serve as potential biomarkers to noninvasively identify AD and VD [178]. Compared with a healthy control group, miR-22*, miR-23a, miR-29a, miR-34b, miR-125b, and miR-130b were upregulated in patients with AD and downregulated in patients with VD by measuring the expression profile of exosomes in serum among patients with AD and VD and healthy controls [178]. Their expression was markedly different between AD and VD groups [178]. These miRNAs could be used as new markers for clinical diagnosis.

EVs are superior to traditional diagnostic biomarkers due to their enhanced stability and diverse molecular composition, showing remarkable auxiliary diagnostic ability in several vascular diseases. Despite their promising diagnostic potential, EVs have primarily been investigated in laboratory settings, and the clinical application of their cargoes is limited. A lack of rapid, convenient, and clinically operable methods for separating EVs hinders their clinical application. However, research on cell-specific EV biomarkers is still lacking for specific diseases because almost all cells in organisms can produce EVs.

### Drug Delivery Vehicles

As drug delivery vehicles, EVs have attracted increasing attention for the treatment and clinical translation of vascular diseases owing to their substantial advantages. First, EVs consisting of lipid bilayers and drugs are packaged inside or loaded onto their surface [179]. It improves drug stability and prevents drug degradation in the body, thereby enhancing the curative effects [180]. Second, EVs can be fused with the cell membrane with good biocompatibility, thereby avoiding the toxicity and side effects of synthetic nanocarriers and maintaining high safety [181]. Third, by modifying EVs carrying recognition molecules, target cells can be recognized and further effectively targeted [182]. Moreover, it is widely available and capable of penetrating various tissue barriers, especially the BBB [183].

Based on current studies, EVs derived from multiple cells have been used as drug delivery vesicles to transport nucleic acids, proteins, chemical drugs, etc., that are crucial in treating vascular diseases such as stroke, myocardial infarction, and cancer. By designing engineered exosomes with the rabies virus glycoprotein peptide on the surface to deliver bioactive nerve growth factor (NGF) mRNA and protein to the ischemic area, which alleviates ischemic brain injury, the level of NGF is increased in the ischemic region [184]. Similarly, brain-derived neurotrophic factor loaded into neural stem cell-derived exosomes were recently shown to considerably improve neurological function and promote tissue repair in MCAO rats [185]. Intriguingly, functionalized EV modification with cardiac-targeting peptide (CTP) and curcumin loaded into EVs to form CTP-EVs-Cur enhanced the bioavailability and cardiac-targeting ability of curcumin. Furthermore, loading miR-144-3p, the main influencing molecule for curcumin therapeutic effects, into CTP-EVs-Cur enhanced the cardioprotective effects under in vitro and in vivo conditions, suggesting that using CTP-EVs to co-deliver curcumin and miR-144-3p could improve the curative effects against myocardial infarction [186]. Huang et al. designed and prepared c(RGDyK)-modified and maternally expressed gene 3 (MEG3)-loaded exosomes (cRGD-Exo-MEG3), which were efficiently delivered to osteosarcoma cells for anti-osteosarcoma roles in vitro and in vivo [187]. Additionally, sEVs were hybridized with synthetic liposomes in a study on breast cancer to create hybrid exosomes (HE), which were then loaded with water-soluble adriamycin. Drug-loaded HE can increase the cytotoxicity in breast cancer cells and amplify the release of pH-sensitive drugs under acidic conditions, making it promising for antitumor drug delivery [180].

## Challenges and Prospects

Currently, studies on EVs remain mostly in the preclinical stage, and EV development in clinical applications still exists unsolved issues.

Isolation methods for EVs do not meet the requirements of EV heterogeneity. Notably, EVs of the same origin may play different roles in cell–cell crosstalk in different cells and vascular diseases. Therefore, the functions and cargoes of EVs are complex and diverse. For example, miR-125b-5p from MSC-EVs alleviates inflammation by inhibiting TLR4 in IS, whereas it reduces autophagy by inhibiting Keap1 in ALI [47, 157]. Jiang et al*.* have provided evidence that miR-1 from hepatocyte-derived EVs aggravates endothelial inflammation and induces cardiovascular diseases [188]. However, in a recent study, miR-1 was shown to directly target CXCR4 to counteract lung cancer growth and metastasis [189]. The different EV effects may originate from their different targets, which may explain why some vascular diseases interact with each other. To solve this problem, establishing standardized techniques to distinguish and isolate different EV subpopulations is essential [190]. Moreover, the reasons for the detected change in the expression level of a single cargo, including the altered paracrine activity of EVs and the altered expression level of cargoes, should be elucidated [191].

Another problem is that most cargo studies have focused on miRNAs, and other cargoes, such as proteins, require further research. Most researchers overexpress specific miRNAs in the cells by transferring plasmids to study intercellular communication mediated by EV miRNAs [192]. Nonetheless, one study has shown that EVs carry only a few miRNAs whose molar concentration is much lower than the assumed functional level. Whether the minority of miRNAs in EVs mediate intercellular communication remains controversial. Considering the current research level, quantifying the miRNA content in EVs is challenging [193, 194]. MSCs are the most popular exogenous EV source for treating vascular diseases [195]. The proteins contained in MSC-derived exosomes at therapeutic doses trigger corresponding biological reactions; however, the miRNA content may not be sufficient to trigger this effect [196]. Thus, specific cargo research in vascular diseases should not be limited to miRNAs, and whether miRNAs or other cargoes at sufficient doses produce effective biochemical effects must be urgently determined.

Additionally, most studies have focused on the mutual interactions between two cell types; however, interactions among an organism as a whole should be considered. The concepts of “neurovascular unit” and “tumor microenvironment” have recently emphasized that current studies should focus on organism integrity [197, 198]. Nerves, vascular systems, and tumor cells are closely related to the surrounding environment [199, 200]. Although EV research is more holistic than traditional research on vascular diseases, its integrity remains insufficient. The interaction between cells should not be limited to two cell types but should be studied between multiple cells, which is a great challenge. Additionally, elucidating the roles of all cargoes in EVs is challenging, and most researchers only pay close attention to the cargoes of interest, ignoring other potentially valuable content. Differential miRNA expression in tissues, cells, serum, plasma, and other samples has been detected by miRNA sequencing or microarray chip technology, and a single miRNA has been selected for subsequent research [201–204]. However, the mechanisms of other miRNAs and the synergistic mechanisms of multiple miRNAs have not been revealed. A thorough elaboration of the relationship between the donor and various receptor cells requires further investigation. Furthermore, systematic research on EVs is required. Revealing these relationships will find more therapeutic targets and disease diagnostic hallmarks, eventually promoting therapeutic drug development for vascular diseases.

Despite the clinical application of EVs facing many problems that need to be addressed, these limitations and challenges have unmasked the unexpected complexity of EV regulatory mechanisms, and inspiring advances have been achieved. The physiological and pathological mechanisms that EVs regulate in vascular diseases, including cerebrovascular diseases, cardiovascular diseases, diabetic complications, and tumors, have been extensively reported. These preclinical results are gradually entering the clinical stage for verification. Several clinical trials have investigated the roles of EVs in vascular diseases. For instance, a completed clinical trial indicated that an additional therapeutic mechanism of ticagrelor against AMI is the inhibition of EV release from activated platelets (*NCT02931045*). Another clinical trial sponsored by Fondazione Don Carlo Gnocchi Onlus aimed to detect blood EVs in patients with stroke using Surface Plasmon Resonance imaging-based biosensors to obtain new prognostic biomarkers in them. This work is ongoing, and we will await the results (*NCT05370105*). Fortunately, some institutions have focused on specific EV cargoes, such as miR-124, micro A, and HSP70, as potential biomarkers of vascular diseases in clinical trials *NCT03384433*, *NCT03542253*, and *NCT02662621*. Although some trials have not yet been completed, these are breakthroughs in the use of specific EV cargoes as clinical markers for vascular diseases.

## Conclusions

Given the prominence of EVs in vascular disease development and progression, several cutting-edge preclinical studies have identified encouraging therapeutic targets and diagnostic biomarkers for vascular disease treatment. These breakthroughs have paved the way for clinical research and have provided new strategies for the drug delivery system. EVs as cell-free therapies are believed to play an essential role in future clinical applications.

## Data Availability

Not applicable.

## Abbreviations

3’ UTRs: 3’ Untranslated regions
ACE 2: Angiotensin-converting enzyme 2
ACE: Angiotensin-converting enzyme
ADSC: Adipose-derived stem cell
AIS: Acute phase IS
AKI: Acute kidney injury
ALI: Acute lung injury
AMI: Acute myocardial infarction
AML: Acute myeloid leukemia
BBB: Blood-brain barrier
BMSC: Bone marrow mesenchymal stem cell
CAF: Cancer-associated fibroblast
CLL: Chronic lymphocytic leukemia
CircRNA: Circular RNA
CMEC: Cardiac microvascular endothelial cell
CML: Chronic myeloid leukemia
CNS: Central nervous system
CRC: Colorectal cancer
CSVD: Cerebral small vascular disease
CTP: Cardiac targeting peptide
CXCR4: C-X-C chemokine receptor type 4
DCM: Diabetic cardiomyopathy
DFU: Diabetic foot ulcers
DLBCL: Diffuse large B cell lymphoma
DN: Diabetic nephropathy
DR: Diabetic retinopathy
EC: Endothelial cell
EGFR: Epidermal growth factor receptor
EPC: Endothelial progenitor cell
EphA2: Ephrin type-A receptor 2
ESC: Embryonic stem cell
EV: Extracellular vesicle
sEV: Small extracellular vesicle
FNDC5: Fibronectin type III domain containing 5
HCC: Hepatocellular carcinoma
HE: Hybrid exosomes
HemEC: Hemangioma endothelial cell
HemSC: Hemangioma stem cell
HF: Heart failure
HIS: Hyperacute phase IS
HNSC: Hippocampal neural stem cell
HUVEC: Human umbilical vein endothelial cell
ICH: Intracerebral hemorrhage
ILV: Intraluminal vesicle
IS: Ischemic stroke
KLF2: Krüppel-like factor 2
KLF4: Krüppel-like factor 4
LOXL4: Lysyl oxidase-like 4
MAPK: Mitogen-activated protein kinase
MCAO: Middle cerebral artery occlusion
MEG3: Maternally expressed gene 3
MHC I: Major histocompatibility complex class I
MHC II: Major histocompatibility complex class II
MIAT: Myocardial infarction associated transcript
MIRI: Myocardial ischemia–reperfusion injury
MiRNA: MicroRNA
MSC: Mesenchymal stem cell
MSC-EVs: MSC-derived EVs
Mst1: Mammalian sterile 20-like kinase 1
mTOR: Mammalian target of rapamyci
MVB: Multivesicular body
NF-κB: Nuclear transcription factor-kappaB
NGF: Nerve growth factor
NLRP3: NOD-like receptor family pyrin domain containing 3
Nrf2: Nuclear factor-erythroid factor 2
NSCLC: Non-small cell lung cancer
OCEM: Ovarian cancer EV miRNA
OPC: Oligodendrocyte precursor cell
PMVEC: Pulmonary microvascular endothelial cell
PTEN: Phosphatase and tensin homolog deleted on chromosome 10
REC: Retinal endothelial cell
RIS: Recovery phase IS
RMEC: Retinal microvascular endothelial cell
RPE: Retinal pigment epithelium
SIRPα: Signal regulatory protein α
SIS: Subacute phase IS
SMC: Subendothelial smooth muscle cell
TEC: Tubular epithelial cell
TLR4: Toll-like receptor 4
TME: Tumor microenvironment
tPA: Tissue plasminogen activator
VD: Vascular dementia
VEGF: Vascular endothelial growth factor
VEGFA: Vascular endothelial growth factor A
VEGFR: Vascular endothelial growth factor receptor
VSMC: Vascular smooth muscle cell
